# A Novel Gene Delivery Vector of Agonistic Anti-Radioprotective 105 Expressed on Cell Membranes Shows Adjuvant Effect for DNA Immunization Against Influenza

**DOI:** 10.3389/fimmu.2020.606518

**Published:** 2020-12-22

**Authors:** Tatsuya Yamazaki, Mrityunjoy Biswas, Kouyu Kosugi, Maria Nagashima, Masanori Inui, Susumu Tomono, Hidekazu Takagi, Isao Ichimonji, Fumiaki Nagaoka, Akira Ainai, Hideki Hasegawa, Joe Chiba, Sachiko Akashi-Takamura

**Affiliations:** ^1^ Department of Microbiology and Immunology, School of Medicine, Aichi Medical University, Aichi, Japan; ^2^ Department of Biological Science and Technology, Tokyo University of Science, Tokyo, Japan; ^3^ Department of Pathology, National Institute of Infectious Diseases, Tokyo, Japan

**Keywords:** antibody gene-vector delivery, adjuvant, RP105, DNA immunization, agonistic antibody, targeting antigen to B cells, cell membrane, influenza

## Abstract

Radioprotective 105 (RP105) (also termed CD180) is an orphan and unconventional Toll-like receptor (TLR) that lacks an intracellular signaling domain. The agonistic anti-RP105 monoclonal antibody (mAb) can cross-link RP105 on B cells, resulting in the proliferation and activation of B cells. Anti-RP105 mAb also has a potent adjuvant effect, providing higher levels of antigen-specific antibodies compared to alum. However, adjuvanticity is required for the covalent link between anti-RP105 mAb and the antigen. This is a possible obstacle to immunization due to the link between anti-RP105 mAb and some antigens, especially multi-transmembrane proteins. We have previously succeeded in inducing rapid and potent recombinant mAbs in mice using antibody gene-based delivery. To simplify the covalent link between anti-RP105 mAb and antigens, we generated genetic constructs of recombinant anti-RP105 mAb (αRP105) bound to the transmembrane domain of the IgG-B cell receptor (TM) (αRP105-TM), which could enable the anti-RP105 mAb to link the antigen *via* the cell membrane. We confirmed the expression of αRP105-TM and the antigen hemagglutinin, which is a membrane protein of the influenza virus, on the same cell. We also found that αRP105-TM could activate splenic B cells, including both mature and immature cells, depending on the cell surface RP105 *in vitro*. To evaluate the adjuvanticity of αRP105-TM, we conducted DNA immunization in mice with the plasmids encoding αRP105-TM and hemagglutinin, followed by challenge with an infection of a lethal dose of an influenza virus. We then obtained partially but significantly hemagglutinin-specific antibodies and observed protective effects against a lethal dose of influenza virus infection. The current αRP105-TM might provide adjuvanticity for a vaccine *via* a simple preparation of the expression plasmids encoding αRP105-TM and of that encoding the target antigen.

## Introduction

Passive immunization using monoclonal antibodies (mAbs) is an important prophylactic and therapeutic method for a variety of diseases such as infections, cancer, and autoimmune diseases (1). Some of the most promising immunotherapy strategies that utilize the self-immune response include immune checkpoint inhibitors, chimeric antigen receptor T cells, and bispecific antibodies (2). Therefore, current passive immunization no longer solely involves neutralizing against pathogens such as canonical passive immunization, which dates back to the production of anti-tetanus and anti-diphtheria serums in the late 19th century by Kitasato and Behring (3). Recently, Clark and colleagues reported a novel passive immunization providing an “adjuvant effect”; they demonstrated that compared to the use of alum, a well-known classical adjuvant, direct conjugation of the antigen to the anti-radioprotective 105 (RP105) mAb could induce more rapid and potent antibody response (4).

The molecule RP105 (CD180) is an orphan Toll-like receptor (TLR) (5, 6). It has a short cytoplasmic tail that lacks the Toll-IL-1 receptor (TIR) domain, which is necessary for mediating TLR signaling (7–9). RP105 is mainly expressed on B cells, dendritic cells, and macrophages (10). It forms a complex with myeloid differentiation protein 1 (MD-1) (11), which is an essential soluble protein for RP105 to be expressed on the cell membrane (12). The agonistic antibody, anti-RP105 mAb, can cross-link RP105 on B cells, followed by the proliferation and activation of B cells (13, 14). In a previous report, Clark and colleagues found that anti-RP105 mAb induces a general and rapid increase in the levels of serum antibodies of all classes except IgG2b and IgA (9, 15). Furthermore, they demonstrated that targeting antigens to RP105 could induce the production of specific antibodies independent of T cells by using CD40-deficient mice (4). They also demonstrated that their strategy could promote traditional 1 (T1) B cells, which are traditional cells during differentiation from immature to mature B cells (16, 17), to induce specific antibodies (18, 19). These results suggest that the anti-RP105 mAb can induce a unique antibody against an antigen, compared with a conventional vaccine, which generally targets mature naïve B cells depending on T cells (20–22).

However, the adjuvant effect requires an anti-RP105 mAb to link the antigen covalently; separated anti-RP105 mAb and antigen cannot significantly enhance the expression of specific antibodies (4). This is a possible obstacle to conducting immunizations because some antigens, such as multi-transmembrane proteins (e.g., G-protein coupled receptors (GPCR), ion channels), would be difficult to covalently link to anti-RP105 mAb, in addition to purifying full-length immunogens (23). To solve the latter problem, our group has conducted DNA immunization and succeeded in obtaining some mAbs against GPCR (24, 25). One significant advantage of DNA immunization is the easy generation and manipulation of antigens using an expression vector (26). In the current study, to simplify vaccination using anti-RP105 mAb as an adjuvant, we evaluated the adjuvant effect of anti-RP105 mAb using DNA immunization, with hemagglutinin (HA), of the influenza virus as a model of membrane proteins. Seasonal influenza continues to be a major health concern because the influenza virus causes annual epidemics and occasional pandemics around the world. Conventional vaccines do not provide complete protection because of antigenic drift and antigenic shift, mainly caused by membrane proteins such as HA, permitting the virus to escape host immunity (27). To control circulating influenza viruses, the development of another vaccine, which can induce potent and broad protection against the virus, is indispensable.

To obtain a potent level of recombinant anti-RP105 mAb (αRP105) into the body, an induction procedure is essential. In our previous study, we succeeded in inducing stable and highly neutralizing (>10 µg) antibodies into the body by electroporation (EP) of a plasmid encoding the mAb in mouse muscles (28). A single use of this method resulted in a long-term prophylactic efficacy before a lethal dose of influenza virus infection. In further studies, we performed hydrodynamic injections (HDs) that involved the rapid injection of a large volume of a plasmid-DNA solution into mice *via* the tail vein (29, 30) and demonstrated that HD could induce rapid and potent neutralizing mAbs (31). A single use of this method resulted in therapeutic efficacy after a lethal dose of influenza virus infection. We also proposed that the new passive immunization using the plasmid encoding the neutralizing mAb could overcome some obstacles of antibody drugs, including high cost and limited supply.

In the current study, to simplify the covalent link of anti-RP105 mAb to the antigen (4), we generated αRP105 bound to the transmembrane domain of the IgG-B cell receptor (TM) (αRP105-TM), which enabled the localization of αRP105-TM and the antigen (i.e., HA) to the cell membrane and may contribute to the linking of anti-RP105 mAb to HA *via* the cell membrane. We detected the co-expressed αRP105-TM and HA on the same cell surface using flow cytometry analysis. We then confirmed that αRP105-TM could stimulate both mature and immature B cells, depending on RP105 expression on the cell surface. We performed a single-dose HD with the plasmids encoding αRP105-TM and HA into BALB/c mice and obtained partial but significant levels of antigen-specific IgG and IgM 14 days after immunization. We also demonstrated the protective effect of this regimen against a lethal dose of influenza virus infection. We then obtained the adjuvant effect of anti-RP105 mAb with a simple preparation of a plasmid encoding the membrane antigen and that encoding αRP105-TM. DNA immunization has mainly focused on the T-cell immune response; that is, the induction of high-quality B cell responses has almost been ignored in the field of DNA immunization (23). To our knowledge, this is the first study to demonstrate that passive immunization with an agonistic antibody bound to TM induces an adjuvant effect. Therefore, our novel passive immunization method could provide a new strategy for DNA immunization that mainly targets B cell activation.

## Materials and Methods

### Plasmid Constructions

**Figure 1:**
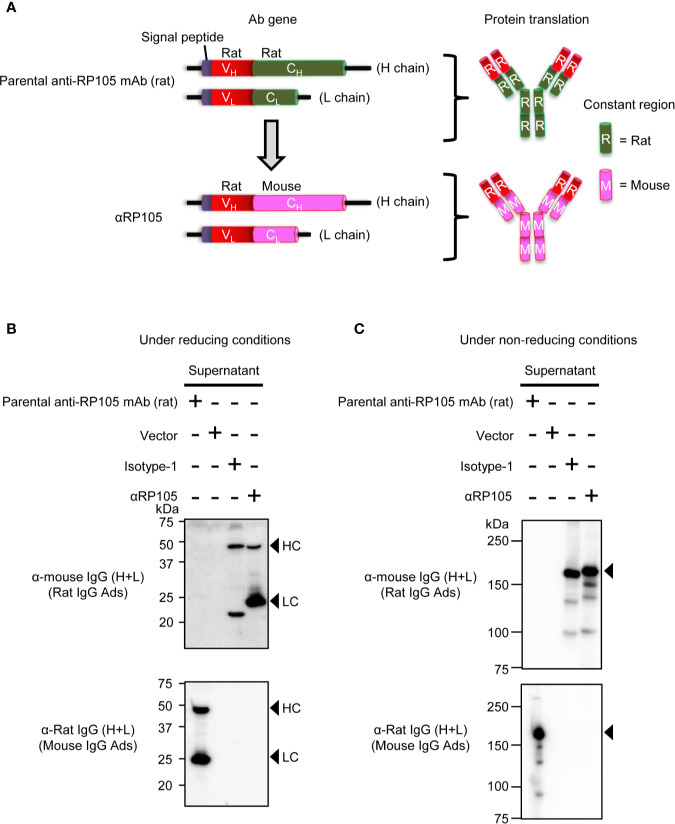
*In vitro* expression of recombinant anti-RP105 (αRP105) from the Ab gene encoding the variable region (V) of anti-RP105 mAb combined with the constant region (C) in mice. **(A)** The genetic construction of recombinant anti-RP105 mAb (αRP105). **(B**, **C)** HEK293T cells were transfected with pCADEST1-empty (Vector), pCADEST1-anti-HA mIgG1 and pCADEST1-anti-HA mkappa (Isotyope-1), or pCADEST1-anti-RP105 mIgG1 and pCADEST1-anti-RP105 mkappa (αRP105). The supernatants were collected after 7 days. The supernatants and parental anti-RP105 mAb (rat) were detected by western blotting under reducing **(B)** or non-reducing **(C)** conditions, followed by probing with HRP-conjugated goat anti-mouse IgG adsorbed rat IgG or HRP-conjugated goat anti-rat IgG adsorbed mouse IgG. The indicated data are representative of two independent experiments.

In our previous report (28), we generated plasmids encoding the genes for the heavy chain (HC; mouse, IgG1) and light chain (LC; mouse, κ) of a neutralizing anti-HA mAb (32), which were used as an isotype control (Isotype-1) in the current study. We also generated another isotype control (Isotype-2), as previously described (28). In brief, total RNA was obtained from mouse hybridoma cells secreting mAbs against chicken ovalbumin (OVA) (unpublished) using the RNeasy kit (Qiagen, Valencia, CA, USA). cDNA from the variable regions, including the signal peptide sequences, was amplified using Ig-primer sets (Novagen, Madison, WI, USA), as previously described. The sequences of the variable regions of the HC (V_H_) and anti-OVA kappa were optimized for expression in mouse cells by FASMAC Co. Ltd. (Kanagawa, Japan). To generate the plasmid encoding anti-RP105 mAb, total RNA was obtained from rat hybridoma cells (RP/14) secreting mAbs against mouse RP105 (14), and cDNA was amplified using a SMART™ RACE cDNA Amplification Kit (Clontech, Mountain View, CA, USA) according to the manufacturer’s instructions. Each construct encoding anti-OVA mAb (Isotype-2) or anti-RP105 mAb (**Figure 1A**) was generated by combining the HC (mouse, IgG1) and LC (mouse, κ) from Isotype-1 by overlap PCR using the primers indicated in **Table S1** in the **Supplementary Material**. The constructs encoding Isotype-2-TM or αRP105-TM were also generated by combining the constant region containing the TM, which removed the encoding region at the C-terminal end of the secreted HC (Accession number: D78344, IMGT, http://www.imgt.org/) (**Figure 4A**), by overlap PCR. The sequence combined a furin cleavage site and foot-and-mouth-disease virus 2A sequence (F2A) (33), and the cDNA encoding Isotype-2-TM or that encoding αRP105-TM were assembled by overlap PCR, as indicated in **Figure 4A**. To generate the construct encoding HA [A/Puerto Rico/8/34 (A/PR8); H1N1], total RNA was isolated from human embryonic kidney (HEK) 293T cells infected with influenza A virus (A/PR8) using TRIzol (Life Technologies, Carlsbad, CA, USA) according to the manufacturer’s instructions. cDNA was generated using SuperScript III Reverse Transcriptase (Invitrogen™ Life Technologies, Carlsbad, CA, USA). The sequence data are described in **Data S1** in the **Supplementary Material**. All constructs were based on the pCADEST1 vector, which was constructed from pCA5, a CAG promoter-driven plasmid, and pDEST12.2 (Invitrogen) (34), and were purified using NucleoBond kits (Clontech) according to the manufacturer’s instructions.

### Mice

BALB/c (8–11 weeks old) (SLC, Shizuoka, Japan), BALB/c RP105^-/-^ (8–14 weeks old), and BALB/c MD-1^-/-^ mice (18 weeks old) (35), backcrossed to BALB/c mice for five to six generations, were maintained under specific pathogen-free conditions. Nude mice (8–11 weeks old) were purchased from SLC. Animal experiments were conducted in accordance with the approval of the Animal Research Committee of Aichi Medical University.

### Antibodies and Reagents

**Figure 2:**
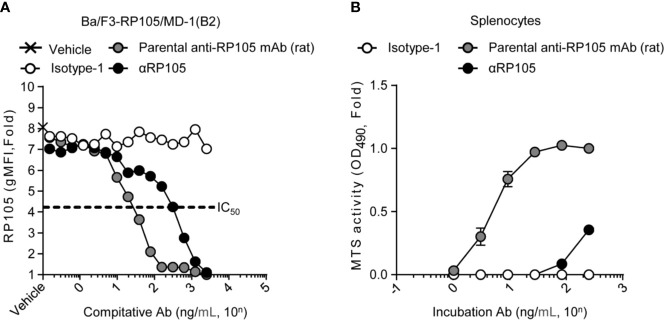
αRP105 can also induce proliferation of splenocytes, but the activity is decreased. **(A)** Ba/F3 cells expressing RP105/MD-1 (B2) were incubated with the parental anti-RP105 mAb (rat), or the supernatant obtained from HEK293T cells expressing anti-HA (Isotype-1) or αRP105 after dilution from 2.5 × 10^3^ ng/ml, as indicated. Then, the cells were incubated with biotinylated parental anti-RP105 mAb (rat), followed by incubation with APC-conjugated streptavidin. Data were obtained using a BD LSRFortessa. Dose-dependent inhibitions are indicated as fold change normalized to geometric mean fluorescence (gMFI) from the incubation with 2.5 × 10^3^ ng/ml of parental anti-RP105 mAb (rat). The IC_50_ was determined by the average of the gMFI obtained from Isotype-1. **(B)** Splenocytes obtained from BALB/c mice were incubated with parental anti-RP105 mAb (rat), Isotype-1, or αRP105 after dilution from 2.5 × 10^2^ ng/ml, as indicated, for 3 days. The proliferation and viability were assessed by an MTS assay. Data are indicated as the mean ± S.D. as fold change normalized to the absorbance at 490 nm from the incubation with 2.5 × 10^2^ ng/ml of parental anti-RP105 mAb (rat). All indicated data are representative of at least two independent experiments.

We purchased HRP-conjugated goat anti-mouse IgG (H+L)-adsorbed rat IgG (Southern Biotech, Birmingham, AL, USA), goat anti-rat IgG (H+L)-adsorbed mouse IgG, goat anti-mouse IgG1, and LC (kappa) binding protein (Santa Cruz Biotechnology) for western blotting. We also purchased HRP-conjugated goat anti-mouse IgG (Southern Biotech) and goat anti-mouse IgM to detect antigen-specific antibodies *via* ELISA. Allophycocyanin (APC)-conjugated goat anti-mouse IgG(H+L) (Southern Biotech), FITC-conjugated rat anti-mouse IgG1 (RMG1-1; BioLegend, San Diego, CA, USA), APC-conjugated rat anti-mouse IgD (11-26c.2a), PerCP-conjugated or PE-conjugated rat anti-mouse B220 (RA3-6B2), biotinylated rat anti-mouse CD86 (GL-1), APC-conjugated streptavidin, PE-conjugated streptavidin, and FITC-conjugated rat anti-mouse IgM (eB121-15F9; eBioscience, San Diego, CA, USA) were purchased. We prepared biotinylated mouse anti-HA IgG1 (31), parental anti-RP105 mAb (rat), mouse anti-MD-1 (JR7G1) (35), and unconjugated anti-HA IgD (31). The parental anti-RP105 mAb (rat) was obtained from the supernatant of the hybridoma (14) (**Figures 1B, C**) or purified from ascites fluid, which was obtained from pristane (Funakoshi, Tokyo, Japan)-primed nude mice that were intraperitoneally inoculated with the hybridoma (36, 37) using the caprylic acid (Wako, Tokyo, Japan)-ammonium sulfate (Katayama Kagaku, Osaka, Japan) precipitation method (38) (**Figures 2A, B**). The concentrations of the parental anti-RP105 mAb (rat) and αRP105 were determined by quantitative ELISA using goat anti-mouse Ig (Southern Biotech) and HRP-conjugated goat anti-mouse IgG (Southern Biotech) as described in the ELISA section.

### Antibody Expression *In Vitro*

HEK293T cells were maintained in 6-well plates in Dulbecco’s modified Eagle’s medium supplemented with 10% heat-inactivated fetal calf serum and penicillin–streptomycin–glutamine (Thermo Fisher Scientific, Waltham, MA, USA). HEK293T cells were transfected with the indicated plasmid vector (2 µg per well) using FUGENE HD Transfection Reagent (Promega, WI, USA) according to the manufacturer’s instructions.

### Western Blotting

The supernatants and the cells, which were lysed with 0.3 ml of lysis buffer [150 mM NaCl, 250 mM Tris-HCl (pH 8.0), 1% NP-40, 0.1% SDS, and 1 × cOmplete (Roche Diagnostics, Mannheim, Germany)], were separated by SDS-PAGE (6 or 12%) under non-reducing or reducing conditions, followed by transfer to a PVDF membrane (Immobilon-P; Merck Millipore). The membrane was blocked with Blocking One reagent (Nacalai Tesque, Tokyo, Japan) for 30 min, followed by incubation at room temperature with the indicated antibodies. The specific bands were visualized using an enhanced chemiluminescence substrate (GE Healthcare UK Ltd., Buckinghamshire, UK) on an ImageQuant LAS4000 system (GE Healthcare). All full-length blots are shown in **Figures S8 and S9**.

### Antibody Expression *In Vivo*

We performed HD (29, 30) using a previously described method (31). Briefly, BALB/c mice and BALB/c RP105^-/-^ mice were injected with a PBS-containing plasmid (e.g., 5 µg/1.6 ml) into the tail vein, where the DNA volume was 8–12% of the body weight. The injection was performed for less than 5 s using a 27-gauge needle. At the indicated times, the serum or spleen was obtained.

### Flow Cytometry Assay

Ba/F3 cells expressing RP105/MD-1 (35) were maintained in RPMI 1640 medium (Thermo Fisher Scientific) containing IL-3 obtained from IL-3-expressing CHO cells, 10% fetal calf serum, 50 µM 2-mercaptoethanol (Nacalai Tesque), and penicillin–streptomycin–glutamine (Thermo Fisher Scientific) and were sorted into clone B2 using a BD FACSAria III (BD Biosciences, San Jose, CA, USA). All flow cytometry analyses were performed using BD LSRFortessa (BD Biosciences) or BD FACSCanto II. Data analyses were performed using FlowJo software version 10.6.1 (FlowJo LLC, Ashland, OR, USA). To analyze the levels of αRP105-TM, HA, RP105, and CD86 on the cells, transfected-HEK293T cells and splenocytes were incubated with APC-conjugated goat anti-mouse IgG (H+L), biotinylated anti-HA IgG1, parental anti-RP105 mAb (rat), or CD86, followed by incubation with APC-conjugated or PE-conjugated streptavidin. Sequential gating for B220^+^, IgM^low^ IgD^high^, and IgM^high^ IgD^low^ were determined using FITC-conjugated rat anti-mouse IgM (eB121-15F9), APC-conjugated rat anti-mouse IgD (11-26c.2a), or PerCP-conjugated or PE-conjugated rat anti-mouse B220 (RA3-6B2). To detect the HEK293T cells co-expressing αRP105-TM and HA, they were incubated with FITC-conjugated anti-mouse IgG1 (RMG1-1) and anti-HA IgD (31), followed by incubation with APC-conjugated rat anti-mouse IgD (11-26c.2a) (**Figure 5A**). To analyze αRP105-TM^+^IgD^+^ B cells, splenocytes were incubated with FITC-conjugated rat anti-mouse IgG1 (RMG1-1), APC-conjugated rat anti-mouse IgD (11-26c.2a) and PE-conjugated rat anti-mouse B220 (RA3-6B2) (**Figure 8**). Single cells were gated using forward scatter-height (FSC-H) versus FSC-width (FSC-W), followed by side scatter (SSC)-H versus SSC-W (39, 40).

### Quantification of αRP105

To determine the concentration of αRP105 in the serum, Ba/F3 cells expressing RP105/MD-1 (B2) were incubated with the obtained serum from mice, followed by incubation with APC-conjugated goat anti-mouse IgG (H+L). The final concentration was determined by the standard curve based on the geometric mean fluorescence intensity (gMFI) of the parental anti-RP105 mAb (rat). The background level of the non-specific reaction was also determined using Ba/F3-null cells. All data analyses were performed using BD LSRFortessa.

### Competitive Binding Assay

A competitive binding assay was carried out as described previously (31) (**Figure S1**). Briefly, Ba/F3 cells expressing RP105/MD-1 (B2) were incubated with the parental anti-RP105 mAb (rat) or the supernatants obtained from HEK293T cells for 30 min at 4°C. Then, they were incubated with biotinylated parental anti-RP105 mAb (rat) followed by incubation with APC-conjugated streptavidin. The binding level was analyzed using BD LSRFortessa.

### Measurement of Spleen Cell Proliferation

Splenocytes were obtained from BALB/c mice treated with red blood cell lysis buffer (17 mM Tris-HCl, 140 mM NH_4_Cl, pH 7.2), followed by incubation at a density of 1.2 × 10^5^ cells/well in 96-well flat-bottomed plates with the parental anti-RP105 mAb (rat) from the ascites fluid or the supernatants, as indicated. Three days later, freshly prepared MTS/PMS solution from Cell Titer 96 Non-Radioactive Cell Proliferation Assay kit (Promega) was added to each well and incubated for 2–4 h. Then, 10% SDS was added to the culture wells to solubilize the formazan product, and the absorbance at 490 nm was recorded using a spectrometer (Spectramax M5, Molecular Devices, CA, USA).

### ELISA

The procedure for measuring the anti-HA antibody, anti-OVA, whole IgG, or IgM levels was as described previously (31, 41, 42). Briefly, a 96-well plate was coated with HA protein, purified from influenza A virus (A/PR8) using an anti-HA antibody-coupled HiTrap NHS-activated HP column (GE Healthcare UK Ltd., Buckinghamshire, England), OVA (Sigma-Aldrich, St. Louis, MO, USA), and goat anti-mouse Ig (Southern Biotech). The plate was incubated with Tris-buffered saline containing bovine casein (Merck Millipore) for blocking. The plate was then incubated with serially diluted supernatants or serum. The cells were then incubated with HRP-conjugated goat anti-mouse IgG (Southern Biotech) or IgM. Finally, expression levels were detected using a TMB solution (Thermo Fisher Scientific). Absorbance at 450 nm was measured using a Spectramax M5. HA-specific IgM antibodies in serum were determined from the standard, which was prepared from the serum obtained from vaccinated mice and expressed as arbitrary units.

### Gross Examination of the Spleen

Four days after HD with pCADEST1-anti-HA mIgG1 and pCADEST1-anti-HA mkappa (Isotyope-1) or pCADEST1-anti-RP105 mIgG1 and pCADEST1-anti-RP105 mkappa (αRP105), the spleens of the mice were obtained, and their weights were measured. Photos were also taken using a camera (Nikon COOLPIX S8100; Nikon, Tokyo, Japan).

### Co-culture of αRP105-TM-Expressing HEK293T Cells and Splenocytes

Two days after transfection with the indicated plasmids, HEK293T cells and splenocytes obtained from BALB/c and littermate-BALB/c RP105^+/-^, RP105^-/-^, MD-1^+/-^, or MD-1^-/-^ mice were co-cultured in complete RPMI 1640 medium for the indicated days. The supernatant was obtained, and the whole IgG level was measured by quantitative ELISA. The splenocytes in the supernatant were also collected and incubated with the indicated antibodies. The expression levels were analyzed using BD LSRFortessa.

### DNA Immunization and Virus Challenge

Female BALB/c mice were subjected to HD with 2.0 ml PBS containing 1.56 µg pCADEST1-HA (A/PR8) and 6.25 µg pCADEST1-anti-RP105 kappa-F2A-anti-RP105 mIgG1-TM. After 14 days, their serum was obtained. The next day, the mice were intranasally infected with a lethal dose of A/PR8 virus (1,000 PFU/50 µl, 40 LD_50_), as previously described (31, 43). Three days post-infection, bronchoalveolar lavage specimens were obtained, and viral titers were determined by a plaque assay. The schedule is shown in **Figure S6**. In another group of animals, survival and weight changes were monitored for 14 days after the virus challenge. After losing 20% of their original body weight, the mice were humanely euthanized (44). A mouse-adapted influenza virus (A/PR8) was grown in the allantoic cavities of 10–11-day-old fertile chicken eggs and stored at −80°C until use.

### Luciferase Assay

A luciferase assay was carried out as described previously (28). Briefly, female BALB/c mice were subjected to HD with 2.0 ml of PBS-containing 1.56 µg pCADEST1-Luc expressing the firefly luciferase gene (28) and 6.25 µg pCADEST1-anti-RP105 kappa-F2A-anti-RP105 mIgG1-TM or pCADEST1-anti-OVA kappa-F2A-anti-OVA mIgG1-TM. One day later, their livers were obtained, cut into pieces, and homogenized in 10 ml of lysis buffer [25 mM Tris/phosphate buffer, 8 mM MgCl_2_ (Wako), 1 mM DTT, 15% glycerol, and 1% TritonX-100 (Boehringer, Mannheim, Germany)]. The specimens were then rotated for 1 h at 4°C. After centrifugation, 10 µL of the supernatant was collected in a black 96-well flat-bottom plate (Corning, Corning, NY, USA), and 50 µL PicaGene Luminescence Kit (Toyo Ink, Tokyo, Japan) was added. Luciferase activity was assessed using a Spectramax M5, and the results were expressed in relative light units (RLU) per protein content (µg) in the supernatant. The protein concentration was measured using a Pierce BCA Protein Assay Kit (Thermo Fisher Scientific) according to the manufacturer’s instructions.

### Neutralizing Assay

The neutralizing titer of the antibody-expressed supernatants was measured by a micro-neutralization assay, as described previously (28, 41). Briefly, the serum was treated with a receptor-destroying enzyme (RDE) (II) (Denka Seiken, Tokyo, Japan) overnight to inactivate nonspecific inhibitors. A total of 100 TCID_50_ of A/PR8 viruses was mixed with an equal volume of RDE-treated serum. The mixtures were then inoculated onto MDCK cells and incubated for three days. The cytopathic effects (CPE) for influenza A virus (A/PR8) infection were evaluated by measuring the absorbance at 630 nm. The neutralization titer was defined as the highest dilution that demonstrated no CPE.

### Plaque Assay

The viral titer was determined by the MDCK-plaque assay, as described previously (31, 45). Briefly, serial 10-fold dilutions of the bronchoalveolar lavage specimens were added to confluent MDCK cells. After 1 h, the inoculum was removed, and 2 ml of agar medium containing acetylated trypsin (Sigma) was overlaid. Two days post-infection, the cells were stained with crystal violet (Nacalai Tesque), followed by counting the number of plaques to determine the viral titer in terms of plaque-forming units (PFU).

### Statistical Analysis

All graphs were constructed using GraphPad Prism 7 (GraphPad Software Inc., San Diego, CA, USA). Data were analyzed using parametric one-way ANOVA, Student’s t-test, non-parametric Mann-Whitney test, and the statistical significance of the survival rate was estimated by a log-rank test (46), where *p* < 0.05 was considered statistically significant.

## Results

### Characterization of the αRP105 mAb *In Vitro*

We previously evaluated the expression of full-length antibodies from the open reading frames encoding mouse anti-HA IgG1 and mouse anti-HA kappa *in vitro* and *in vivo* (28). Anti-RP105, a rat anti-mouse RP105 IgG2a mAb, has been well characterized for its potent agonistic effect on the proliferation of B cells (35, 47), but the characteristics of recombinant anti-RP105 mAb (αRP105) from the antibody gene are unknown. To confirm this, we first cloned the open reading frame from the rat hybridoma producing anti-RP105 mAb (14). To reduce its immunogenicity in mice, we also converted the gene encoding the constant region in both the HC and LC from the parental rat gene sequence to mice (**Figure 1A**) and subcloned them separately into the expression plasmid, pCADEST1. In order to analyze their expression level *in vitro*, we transfected these plasmids into HEK293T cells and analyzed the antibody levels in the supernatants. Under reducing conditions, we observed specific bands of the HC and LC at approximately 50 kDa and 25 kDa (**Figure 1B**, upper panel, lane 4), respectively. We also observed specific bands of the full-length antibodies at around 150 kDa under non-reducing conditions (**Figure 1C**, upper panel, lane 4). On the other hand, we could not detect the band using anti-rat IgG both under reducing and non-reducing conditions (**Figures 1B, C**, lower panel, lane 4). These results suggested that αRP105 could express a full-length antibody containing two HCs and two LCs, which was derived from mouse constant regions, but not from rat constant regions, of the parental anti-RP105 mAb.

We next evaluated the specificity of αRP105 against RP105 using a competitive binding assay (**Figure S1**). Indeed, pre-incubation of Ba/F3 cells expressing RP105/MD-1 with αRP105 inhibited the binding of parental anti-RP105 mAb (rat). It was suggested that αRP105 could recognize the same epitope of RP105, similar to parental anti-RP105 mAb (rat) (**Figure 2A**). However, the IC_50_ of αRP105 was approximately 11-fold higher than that of the parental anti-RP105 mAb (rat). To confirm the agonistic effect of αRP105, we evaluated the level of B cell proliferation by incubating splenocytes with parental anti-RP105 mAb (rat) or αRP105. The results indicated that αRP105 could induce cell proliferation but it exhibited a weaker effect than the parental anti-RP105 mAb (rat): αRP105 required approximately an 80-fold higher concentration to obtain the same level of proliferation as that of parental anti-RP105 mAb (rat) (**Figure 2B**). These results suggested that αRP105 could recognize RP105 and enhance the proliferation of B cells, similar to the parental anti-RP105 mAb (rat), although its effect was much weaker.

### Expression Level of αRP105 mAbs in Mice

**Figure 3:**
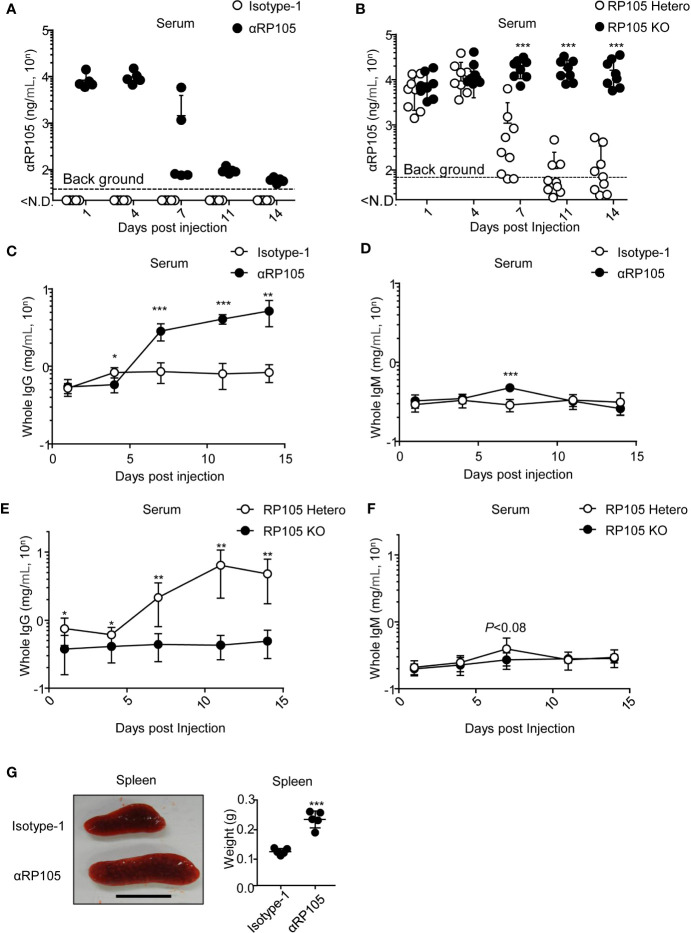
αRP105 enhances the level of whole IgG in the serum, which depends on RP105, and enlarges the spleen. **(A)** BALB/c mice (n = 5) were subjected to HD with pCADEST1-anti-HA mIgG1 and pCADEST1-anti-HA mkappa (Isotyope-1) or pCADEST1-anti-RP105 mIgG1 and pCADEST1-anti-RP105 mkappa (αRP105). After the indicated times, the serum was obtained. The expression level of αRP105 was quantified using Ba/F3 cells expressing RP105/MD-1 (B2) using a BD LSRFortessa. The background level [38.1 (=10^1.581^) ng/ml] was also determined using Ba/F3-null cells. **(B)** BALB/c RP105-Hetero or RP105-KO mice were subjected to HD with pCADEST1-anti-RP105 mIgG1 and pCADEST1-anti-RP105 mkappa. After the indicated times, the serum was obtained. The expression level of αRP105 was quantified by flow cytometry using Ba/F3 cells expressing RP105/MD-1 (B2). The background level [68.4 (=10^1.835^) ng/ml] was also determined using Ba/F3-null cells. **(C**, **D)** Whole IgG **(C)** and IgM **(D)** levels in the serum **(A)** were analyzed by quantitative ELISA. (**E**, **F**) Whole IgG **(E)** and IgM **(F)** levels in the serum **(B)** were analyzed by quantitative ELISA. **(G)** Four days post-HD with the plasmids encoding Isotyope-1 or αRP105 into BALB/c WT mice (n = 5), the spleens were collected, and their weights were measured. The scale bar represents 1 cm. The indicated data **(A, C, D, G)** are representative of at least two independent experiments and are indicated as the mean ± S.D. The indicated data **(B, E, F)** are combined from two independent experiments (n = 8–9) and are indicated as the mean ± S.D. The detection limit was over 0.0147 (=10^-1.832^) mg/ml **(C**, **E)** or 0.0234 (=10^-1.63^) mg/ml **(D**, **F)**. *P < 0.05, **P < 0.01, ***P < 0.001 (Student’s *t-*test).

We previously demonstrated that HD, which involves the rapid injection of a large volume of a DNA solution containing plasmids encoding mAbs into mice *via* the tail vein, could rapidly and potently induce recombinant antibodies in the serum (31). To obtain a potent expression of αRP105 in the body, we also administered HD with plasmids encoding anti-RP105 mAb into BALB/c mice. One and four days post-HD, we detected a similar level of αRP105 in the serum as previously described (31). The αRP105 concentration reached approximately 9,900 (=10^3.996^) ng/ml (**Figure 3A**). However, from 7 days post-HD, the level of αRP105 rapidly decreased until it was not significantly detected at 14 days post-HD. To confirm whether this unstable expression depends on RP105, we also administered HD with the plasmids encoding anti-RP105 mAb into RP105-knockout (KO) mice. Unlike HD in RP105 heterozygous mice, the potent level of the antibodies could be stably detected in RP105-KO mice within at least 14 days post-HD (**Figure 3B**). These results suggested that HD with the plasmid encoding anti-RP105 mAb could induce a potent level of antibodies, but not stably in serum, due to RP105-expressing cells.

### αRP105 mAb Significantly Stimulates IgG-Expressing Cells

In a previous study, Clark and colleagues demonstrated that the administration of a high dose of purified anti-RP105 mAb (250 µg) induced a >15-fold increase in serum IgG and an 11-fold transient increase in serum IgM (15). Here, we examined whether αRP105 could increase polyclonal antibodies in the serum. Seven days post-HD, the level of whole IgG significantly increased (3.3-, 5.1-, and 6.2-fold average increase at days 7, 11, and 14, respectively) (**Figure 3C**). On the other hand, whole IgM also significantly increased at day 7 post-HD (1.6-fold average), but only transiently, similar to previous studies (15); it did not significantly increase at other days (**Figure 3D**). We also confirmed that the increase of whole IgG, but not whole IgM, was significantly dependent on RP105 (**Figures 3E, F**). In a previous study, Clark and colleagues also indicated that the spleen of parental anti-RP105 mAb-treated mice increased 3-fold compared with control mice (15). To confirm this observation, we also administered the plasmid encoding anti-RP105 mAb *via* HD and obtained the spleen 4 days after. Similarly, we also detected an enlarged spleen after treatment with αRP105 (**Figure 3G**, left panel). Its weight was approximately 1.9-fold higher than that of the isotype control (**Figure 3G**, right panel). However, these observations were not significantly different at 14 days post-HD (data not shown). These results suggest that approximately 10 µg of αRP105 induced by HD could also stimulate B cells, resulting in IgG production, followed by an increase in serum levels of IgG and spleen size, but some of these effects were transient.

### Characterization of αRP105 mAbs Bound With TM *In Vitro*

**Figure 4:**
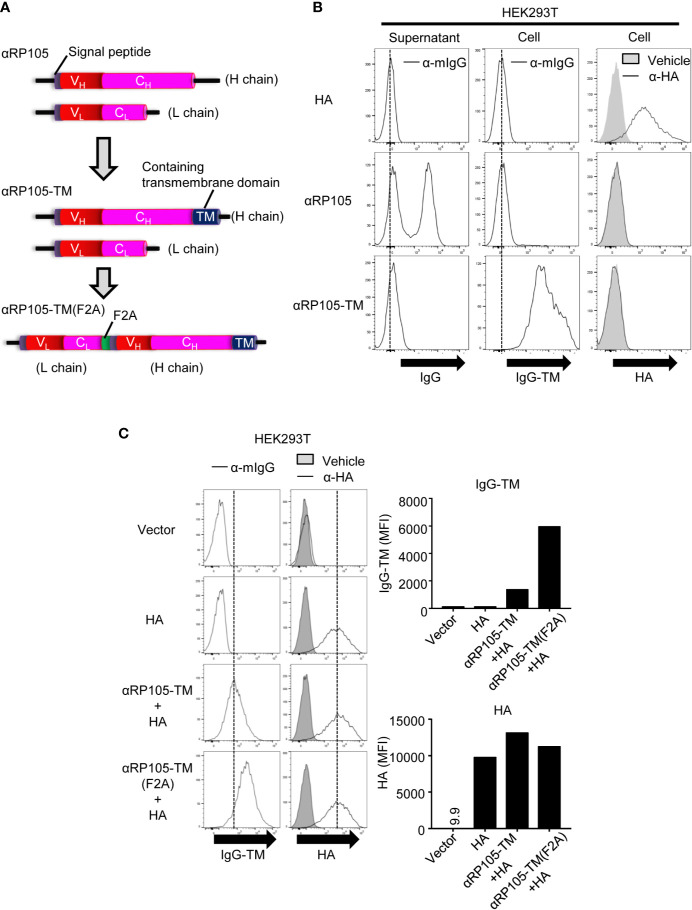
The level of αRP105-TM expression on the cell membrane is enhanced using the F2A element. **(A)** The middle diagram indicates the genetic construction of anti-RP105 mIgG1 bound to the transmembrane (TM). The lower diagram indicates the genetic construction of anti-RP105 mIgG1-TM bound to anti-RP105 kappa *via* the F2A sequence. **(B)** HEK293T cells were transfected with pCADEST1-HA (A/PR8) as a control of membrane protein, pCADEST1-anti-RP105 mIgG1 and pCADEST1-anti-RP105 mkappa (αRP105), or pCADEST1-anti-RP105 mIgG1-TM and pCADEST1-anti-RP105 mkappa (αRP105-TM). Two days later, the supernatants and cells were collected. Ba/F3 cells expressing RP105/MD-1 (Balk) were incubated with the supernatants, followed by incubation with APC-conjugated anti-mouse IgG (Left panel). HEK293T cells were also incubated with APC-conjugated anti-mouse IgG (Middle panel) or biotinylated anti-HA IgG1, followed by incubation with APC-conjugated streptavidin (Right panel). The expression level was analyzed using a BD FACSCanto II. **(C)** HEK293T cells were transfected with pCADEST1-empty (Vector), pCADEST1-HA (A/PR8), and pCADEST1-anti-RP105 mIgG1-TM and pCADEST1-anti-RP105 mkappa (αRP105-TM) or pCADEST1-anti-RP105 kappa-F2A-anti-RP105 mIgG1-TM [αRP105-TM (F2A)]. Two days later, the cells were collected and incubated with APC-conjugated anti-mouse IgG (Left panel) or biotinylated anti-HA IgG1, followed by incubation with APC-conjugated streptavidin (Right panel). The expression level was analyzed using a BD LSRFortessa. All indicated data are representative of at least two independent experiments.

As mentioned above, we succeeded in constructing a plasmid expressing αRP105 (**Figure 4A**, upper construct) and demonstrated its agonistic effectiveness. Furthermore, to mimic the covalent binding between anti-RP105 mAb and the antigen, we first generated a plasmid expressing αRP105 bound to the TM (αRP105-TM), which was expected to be expressed on the cell surface (**Figure 4A**, middle construct). In order to analyze the expression of αRP105-TM *in vitro*, we transfected the plasmids encoding HA, αRP105, or αRP105-TM into HEK293T cells and analyzed the antibody expression in the supernatant and on the cell surface. We could significantly detect αRP105 expression in the supernatant, but not primarily on the cell surface (**Figure 4B**, middle panel). In contrast, αRP105-TM was detected only on the cell surface, but not in the supernatant (**Figure 4B**, lower panel). As shown in **Figure S2A**, we also observed a specific band for αRP105-TM, and the band size of the HC was slightly larger than that of αRP105 (**Figure S2A** upper panel, lanes 2 and 3), while both LCs were observed to be of the same size (**Figure S2A** lower panel, lanes 2 and 3). The size of full-length antibodies was also observed to be slightly larger (**Figure S2B**, lanes 2 and 3). These results suggested that the enlarged size was caused by the additional TM domain. With these, we confirmed the successful construction of αRP105-TM.

A previous study demonstrated that mAbs from the HC bound to the LC *via* furin-2A (F2A) elements obtained stable expression (33). This element has been used for mAb expression *in vivo* (33, 48–50). Several studies have also compared the gene structure for optimizing antibody expression (51, 52); however, it is unknown whether F2A promotes the expression of αRP105-TM. Thus, we constructed a plasmid encoding anti-RP105 mkappa bound to anti-RP105 mIgG1-TM *via* the F2A element [αRP105-TM (F2A)] (**Figure 4A**, lower construct). In order to analyze the expression of αRP105-TM (F2A) *in vitro*, we transfected the plasmids encoding αRP105-TM or αRP105-TM (F2A) into HEK293T cells. As shown in **Figure S2C**, we observed the same-sized band of the HC from αRP105-TM and αRP105-TM (F2A) under non-reducing conditions (**Figure S2C**, upper panel, lanes 2 and 3). In contrast, the band size of the LC from αRP105-TM (F2A) was larger than that of αRP105-TM (**Figure S2C**, lower panel, lanes 2 and 3). Under non-reducing conditions, the size of full-length antibodies was almost the same, at approximately 150 kDa, in both αRP105-TM and αRP105-TM (F2A) (**Figure S2D**, lanes 2 and 3). To analyze whether F2A promotes the expression of αRP105-TM on the cell membrane, we co-transfected the plasmids encoding αRP105-TM (F2A) and HA into HEK293T cells. The expression level of αRP105-TM (F2A) was 4-fold higher than that of αRP105-TM (**Figure 4C**, left panel), whereas the expression of co-transfected HA was almost the same (**Figure 4C**, right panel). These results demonstrated that F2A elements could simplify transfection for the expression of αRP105-TM with one plasmid and also promote expression on the cell membrane.

### Characterization of Another Recombinant mAb Bound With TM (Isotype-2-TM) *In Vitro*

The current study focused on using αRP105 as an adjuvant for influenza HA vaccine. To evaluate the naturally occurring anti-HA antibodies from the vaccine, we prepared another isotype control for αRP105-TM. We cloned another open reading frame from mouse hybridoma producing anti-OVA IgG1 (kappa) (Isotype-2) and subcloned it into pCADEST1. In order to analyze its expression level *in vitro*, we transfected this plasmid into HEK293T cells and analyzed the antibody levels in the supernatant. Under reducing conditions, we detected the specific bands of HC and LC, but their sizes were less than approximately 50 or 25 kDa (**Figure S3A**, lane 3), respectively. We also observed specific bands of full-length antibodies at less than approximately 150 kDa under non-reducing conditions (**Figure S3B**, lane 3). To confirm the expression level *in vivo*, we administered HD with the plasmids encoding Isotype-2 into BALB/c mice. At least 10 days post-HD, we could detect a potent and stable level of Isotype-2 expression in the serum (**Figure S3C**). The level was also approximately 8,700 (=10^3.940^) ng/ml, similar to that of αRP105.

We also constructed Isotype-2 bound to the the TM (Isotype-2-TM). Similar to αRP105-TM, we observed that the band size of the HC was slightly larger than that of Isotype-2 under reducing conditions (**Figure S4A** upper panel, lanes 2 and 3), although its detection required lysis with a buffer containing 2% SDS. Both LCs were observed to be of the same size (**Figure S4A** lower panel, lanes 2 and 3). We also obtained a smeared band of the full-length antibody at over 150 kDa from Isotype-2-TM under non-reducing conditions (**Figure S4B**, lane 3). These data demonstrated that Isotype-2-TM (possessing a TM) could be expressed, but this partial feature was different from that of αRP105-TM.

We also confirmed the expression of Isotype-2 (F2A), similar to αRP105-TM (F2A), although its detection required lysis with a buffer containing 2% SDS (**Figures S4 C and D**). The LC of Isotype-2-TM (F2A) was larger than that of Isotype-2-TM (**Figure S4C**, lower panel, lanes 2 and 3), whereas the HCs were almost of the same size (**Figure S4C**, upper panel, lanes 2 and 3). We also obtained a smeared band of the full-length antibody at over 150 kDa from both Isotype-2-TM and Isotype-2-TM (F2A) under non-reducing conditions (**Figure S4D**, lanes 3 and 4). These results (**Figure S4C**) and those from αRP105-TM (F2A) (**Figures S2C**) suggest that the 2A peptide worked by automatic cleavage to separate the LC and HC, whereas the furin cleavage site did not work because the size of the LC was increased.

### Biological Activity of αRP105-TM mAbs Expressed by F2A Elements

**Figure 5:**
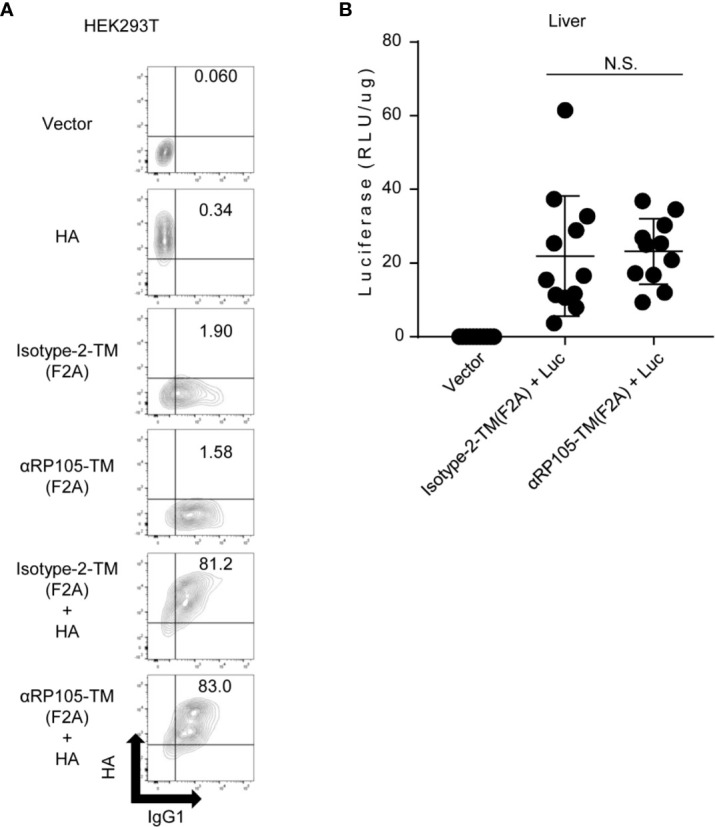
Antigen expression level *in vivo* and *in vitro* is not significantly different with co-expression of Isotype-2-TM and αRP105-TM. **(A)** HEK293T cells were co-transfected as indicated. Isotype-2-TM (F2A) was expressed from pCADEST1-anti-OVA kappa-F2A-anti-OVA mIgG1-TM (**Figure S4**). Two days later, these cells were collected and incubated with FITC-conjugated anti-mouse IgG1 and anti-HA IgD, followed by incubation with APC-conjugated anti-mouse IgD. The indicated numbers represent the ratio (%) of IgG1^+^HA^+^ cells in total. The expression level was analyzed using a BD LSRFortessa. The indicated data are representative of at least two independent experiments. **(B)** Female BALB/c mice were subjected to HD with either pCADEST1-empty (Vector), pCADEST1-anti-OVA kappa-F2A-anti-OVA mIgG1-TM [Isotype-2-TM (F2A)] and pCADEST1-luciferase (Luc), or pCADEST1-anti-RP105 kappa-F2A-anti-RP105 mIgG1-TM [αRP105-TM (F2A)] and pCADEST1-Luc, as indicated. One day later, the liver was obtained, and luciferase activity was determined. The indicated data are combined from three independent experiments (n = 11–12) and indicated as the mean ± S.D. N.S., not significant (Mann-Whitney test).

To confirm the co-expression of HA and IgG-TM on the same cell membrane, we also co-transfected the plasmids encoding HA and either the plasmid encoding αRP105-TM (F2A) or Isotype-2-TM (F2A) into HEK293T cells. To distinguish between the two proteins, IgG-TM and HA, we detected them using anti-mIgG1 and anti-HA IgD, which were constructed in our previous study (31). We then obtained the expression of HA and αRP105-TM (F2A) or HA and Isotype-2-TM (F2A) on the same cell membrane (sixth and fifth panel from the top of **Figure 5A**). The ratio of HA^+^IgG-TM^+^ cells was almost the same between cells co-expressing HA and αRP105-TM (F2A) and those co-expressing HA and Isotype-2-TM (F2A) (83.0 and 81.2%, respectively). We also confirmed the same level of the antigen co-expressing IgG-TM *in vivo*, altering the luciferase-expressing vector from HA (**Figure 5B**). These results suggested that the antigen, HA, and agonistic antibody, αRP105-TM, from the current constructions could be localized on the cell membrane. They also suggested that the localization ratio was almost the same between αRP105-TM (F2A) and Isotype-2-TM (F2A) both *in vitro* and *in vivo*.

### αRP105-TM Stimulates Both Mature and Immature B Cells by Association With RP105 Expressed on the Cell Surface

**Figure 6:**
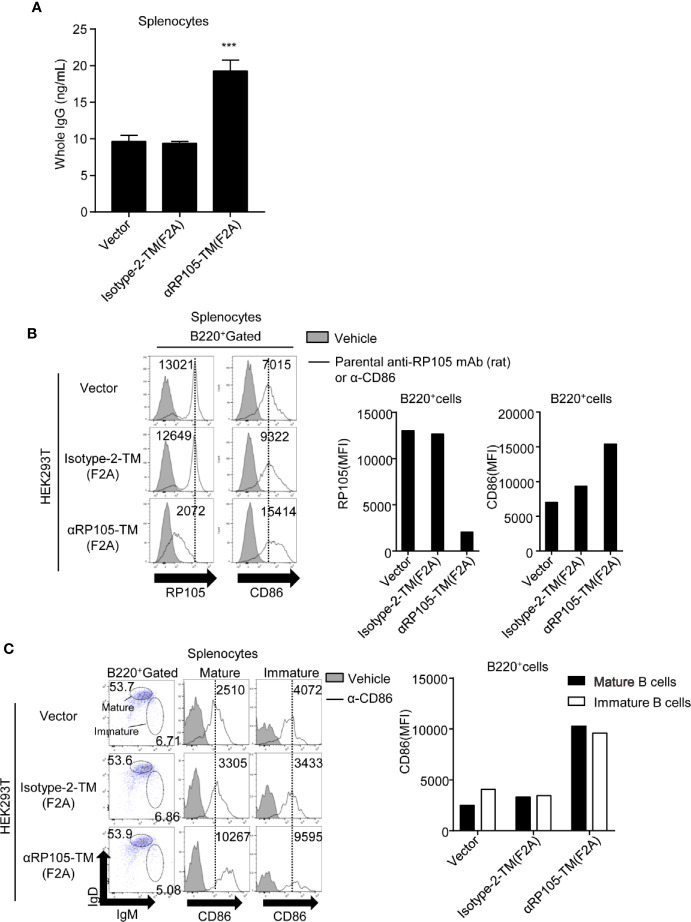
αRP105-TM can activate both mature and immature B cells. **(A)** HEK293T cells were transfected with pCADEST1-empty (Vector), pCADEST1-anti-OVA kappa-F2A-anti-OVA mIgG1/TM [Isotype-2-TM (F2A)], or pCADEST1-anti-RP105 kappa-F2A-anti-RP105 mIgG1-TM [αRP105-TM (F2A)]. Two days later, the splenocytes obtained from BALB/c mice were incubated with the HEK293T cells for 7 days. The supernatant was obtained, and the whole IgG level was measured by quantitative ELISA. The detection limit was over 3.91 ng/ml. ***P < 0.001 (One-way ANOVA). **(B**, **C)** HEK293T cells were transfected as indicated. Two days later, splenocytes obtained from BALB/c mice were co-cultured with HEK293T cells for 2 days. Then, the splenocytes, which were gated on B220 **(B)**, IgM^low^ IgD^high^ as mature B cells (Middle panel in **C**), or IgM^high^ IgD^low^ as immature B cells (Right panel in **C**), were also incubated with biotinylated parental anti-RP105 mAb (rat) or anti-CD86 antibodies as indicated, followed by incubation with PE-conjugated streptavidin. The indicated numbers on each gate (Left panel in **C**) respectively represent the percentage of cells of mature or immature B cells gated on B220^+^ cells. The numbers in the histogram represent MFI. All indicated data are representative of two independent experiments.

Since we confirmed that the plasmid expressing αRP105-TM was localized on the cell membrane, we next investigated the agonistic effect of αRP105-TM. We co-cultured αRP105-TM-expressing HEK293T cells and splenocytes obtained from BALB/c mice. We first measured the whole IgG level in the supernatant. The level from B cells co-cultured with αRP105-TM-expressing cells was approximately 2-fold higher than that in Isotype-2-TM cells expressing (**Figure 6A**). To analyze whether B cells were activated by αRP105-TM, we measured the expression level of the activation marker, CD86, on the B cell surface by flow cytometry. The level of the cells co-cultured with αRP105-TM-expressing cells was approximately 1.7-fold higher than that of Isotype-2-TM cells expressing (**Figure 6B**, right panel, and right bar graph). We also found that the level of RP105 decreased in B cells co-cultured with αRP105-TM-expressing HEK293T cells, compared with those co-cultured with Isotype-2-TM-expressing HEK293T cells (**Figure 6B**, left panel and left bar graph). This result suggests two possibilities: one is the masked epitope by αRP105-TM, and the other is the internalization of RP105 by the association of αRP105-TM as previously described (4). To clarify whether mature or immature B cells were activated by αRP105-TM, we separated them, with IgM^low^ IgD^high^ expressing cells as mature B cells and IgM^high^ IgD^low^ as immature B cells, as previously described (53) (**Figure 6C**, left panel), and measured the level of CD86 in each. The levels of both mature (**Figure 6C**, middle panel) and immature B cells (**Figure 6C**, right panel) co-cultured with αRP105-TM-expressing cells were approximately 3.0-fold higher than that of Isotype-2-TM-expressing cells (**Figure 6C**, bar graph). These results suggested that αRP105-TM could activate both mature and immature B cells, followed by the promotion of IgG production.

**Figure 7:**
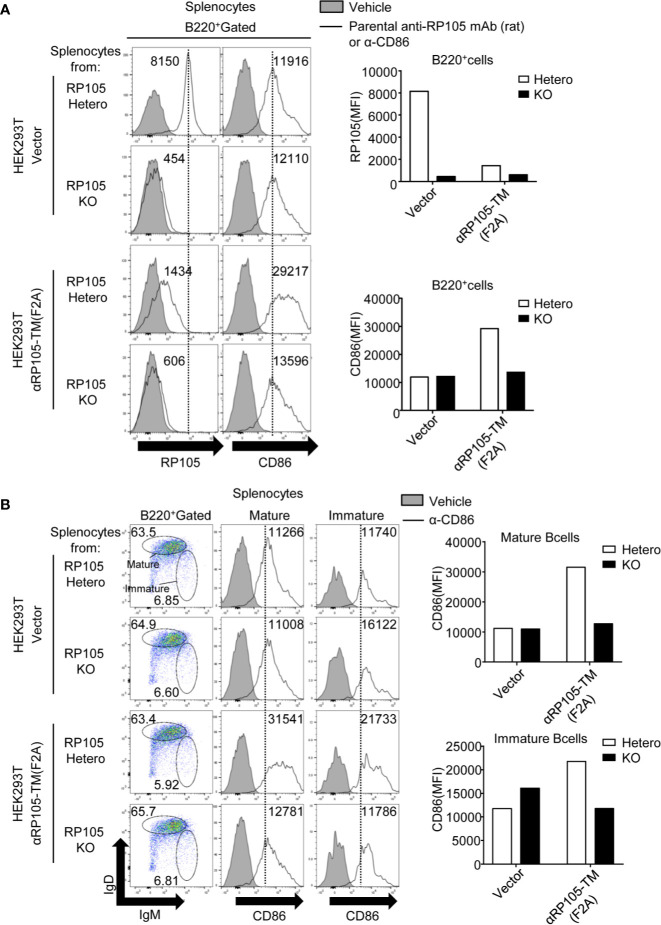
αRP105-TM can activate both immature and mature B cells depending on RP105. **(A)** HEK293T cells were transfected with pCADEST1-empty (Vector) or pCADEST1-anti-RP105 kappa-F2A-anti-RP105 mIgG1-TM [αRP105-TM (F2A)]. Two days later, the splenocytes that were obtained from RP105-Hetero and RP105-KO BALB/c mice were co-cultured with HEK293T cells for 2 days. The splenocytes, which were gated on B220, were also incubated with biotinylated parental anti-RP105 mAb (rat) or anti-CD86 antibodies as indicated, followed by incubation with PE-conjugated streptavidin. **(B)** The splenocytes, which were gated on IgM^low^ IgD^high^ as mature B cells (Middle panel) or IgM^high^ IgD^low^ as immature B cells (Right panel), were also incubated with biotinylated anti-CD86 antibodies, followed by incubation with PE-conjugated streptavidin. The indicated numbers on each gate (Left panel in **B**) respectively represent the percentage of cells of mature or immature B cells gated on B220^+^ cells. The numbers in the histogram represent MFI. The expression level was analyzed using a BD LSRFortessa. All indicated data are representative of two independent experiments.

To analyze whether the B cell activation induced by αRP105-TM depends on RP105, we also co-cultured αRP105-TM-expressing HEK293T cells and splenocytes obtained from RP105-hetero mice or RP105-KO mice. Although the level of CD86 on RP105-expressing B cells (**Figure 7A**, right panel, and lower bar graph) increased similar to that of wild-type mice (**Figure 6B**), that of RP105-deficient cells was the same as that of the vector control (**Figure 7A**, right panel, and lower bar graph). As shown in **Figure 7B**, the levels of CD86 in both the RP105-deficient mature and immature B cells were the same as that of the vector control. Previous reports have demonstrated that MD-1, a soluble protein associated with RP105, is indispensable for the cell surface expression of RP105 (11, 12) (**Figure S5A**, left panel, and left bar graph). To analyze whether αRP105-TM interacts with the cell surface expression of RP105 for the activation of B cells, we co-cultured αRP105-TM-expressing HEK293T cells and splenocytes obtained from MD-1-hetero or MD-1-KO mice. Although the level of CD86 on MD-1-expressing B cells (**Figure S5A**, middle panel, and middle bar graph), which also expressed RP105 on the cell membrane (**Figure S5A**, left panel, and left bar graph), was increased (**Figure S5A**, right panel, and right bar graph), that of MD-1-deficient cells, in which RP105 was not detected, was the same as that of the vector control. As shown in **Figure S5B**, the levels of CD86 in both MD-1-deficient mature and immature B cells were the same as that of the vector control. These results suggested that the activation of B cells with αRP105-TM required RP105 expression on the cell surface.

**Figure 8:**
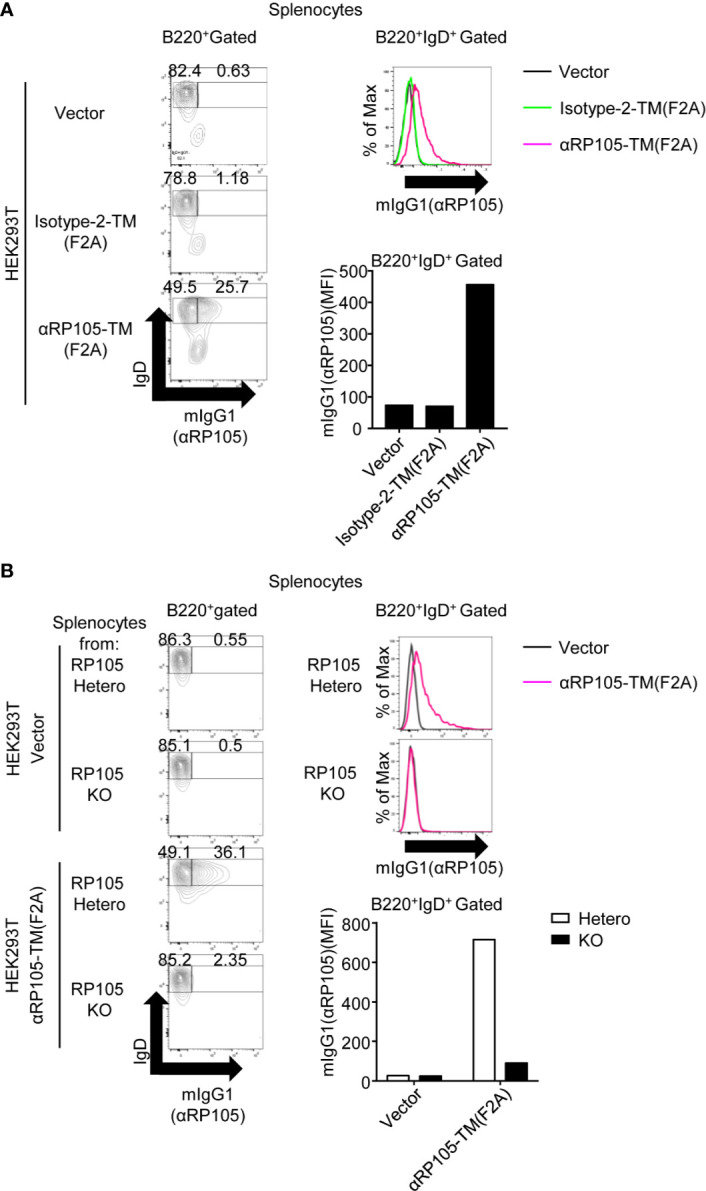
αRP105-TM can associate with the B cell membrane, depending on RP105. **(A)** From **Figures 6B, C**, the splenocytes were collected and incubated with FITC-conjugated anti-mouse IgG1 and APC-conjugated anti-mouse IgD. **(B)** From **Figures 7A, B**, the splenocytes were collected and incubated with FITC-conjugated anti-mouse IgG1 and APC-conjugated anti-mouse IgD. The indicated numbers on each gate in the left panel represents the percentage of cells of IgD^+^mIgG1^-^ or IgD^+^mIgG1^+^ gated on B220^+^ cells, respectively. The histogram and bar graph in the panels on the right represent the level of mIgG1, which represents the level of αRP105 on B220^+^ IgD^+^cells. The expression level was analyzed using a BD LSRFortessa. All indicated data are representative of two independent experiments.

To reveal the interaction between αRP105-TM in HEK293T cells and RP105 on B cells, we analyzed whether the decreased level of RP105 (**Figure 6B**, left panel) was caused by masking with αRP105-TM associated with RP105 on the cell membrane. We collected the splenocytes in the supernatant (**Figures 6B, C**) and measured the level of αRP105-TM on B cells with anti-mIgG1 in the single-cell gated population using flow cytometry. We first confirmed that the natural IgG1 on B cells co-cultured with HEK293T-null cells (vector) was not significantly detected (**Figure 8A**, left panel, first data from the top). Under these conditions, the IgG1-positive B cells were detected in 25.7% of B220-positive cells in the splenocytes (**Figure 8A**, left panel, third data from the top). Approximately 6.5-fold higher levels of IgG1-positive B cells gated on B220-positive and IgD-positive cells were detected in splenocytes co-cultured with αRP105-TM-expressing HEK293T cells than in those co-cultured with Isotype-2-TM-expressing HEK293T cells (**Figure 8A**, right panel and graph). We also confirmed that the interaction of αRP105-TM on HEK293T cells and B cells depended on RP105 (**Figure 8B**). These results suggested that αRP105-TM on HEK293T cells was transferred to the B cell membrane depending on RP105, followed by masking RP105 and blocking another anti-RP105 mAb binding. Overall, they also suggest that B cells were activated by the interaction of αRP105-TM with RP105 and the transfer of molecules.

### Passive Immunization With the Plasmids Encoding αRP105-TM Enhances the Antigen-Specific Antibody Response and Protective Effect Against a Lethal Dose of Influenza Virus Infection in Mice

**Figure 9:**
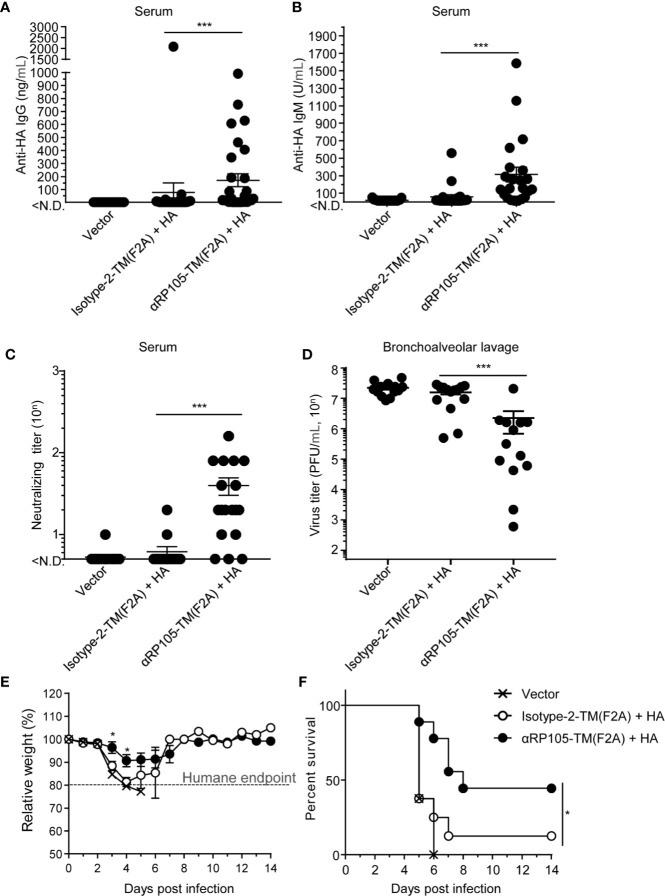
αRP105-TM can significantly increase the level of neutralizing antibodies against the influenza virus and provide protection. **(A–F)** Female BALB/c mice were subjected to HD with either pDEST1-empty (Vector), pCADEST1-anti-OVA kappa-F2A-anti-OVA mIgG1-TM [Isotype-2-TM (F2A)] and pCADEST1-HA (A/PR8), or pCADEST1-anti-RP105 kappa-F2A-anti-RP105 mIgG1-TM [αRP105-TM (F2A)] and pCADEST1-HA (A/PR8). Fourteen days later, the serum was obtained, and the level of anti-HA IgG **(A)** and that of anti-HA IgM **(B)** were measured by quantitative ELISA. The detection limit was over 1.2 ng/ml **(A)** or 15.6 U/ml **(B)**. **(C)** The neutralizing titer against the influenza virus was also determined by a micro-neutralization assay. The detection limit was over 10. **(D)** The next day, the mice were infected with a lethal dose of A/PR8 virus (1,000 PFU). Three days post-infection, the bronchoalveolar lavage specimens were obtained, and viral titers were determined by a plaque assay. **(E, F)** The body weight **(E)** and survival rates **(F)** in another group were monitored for 14 days. The body weight was expressed relative to the initial mean body weight of each group. **(A–D)** The indicated data are combined either from four independent experiments (n = 27–29) **(A)**, three independent experiments (n = 21–22) **(B)**, two independent experiments (n = 17–18) **(C)**, or two independent experiments (n = 13–14) **(D)**. **(E**, **F)** The indicated data are representative of three independent experiments. All error bars represent the S.E.M. *P < 0.05, ***P < 0.001 [**(A**–**E)**, Mann-Whitney test; **(F)**, Log-rank test].

To evaluate whether αRP105-TM enhanced specific antibody responses as a molecular adjuvant in DNA immunization, we conducted HD with plasmids encoding HA or αRP105-TM in mice. Fourteen days later, we measured the levels of specific IgG and IgM antibodies against HA in the serum. We could not detect significant amounts of specific antibodies in mice that were immunized with HA and Isotype-2-TM (**Figures 9A, B**). We observed that approximately 31% of the mice which were immunized with HA and αRP105-TM induced specific IgG (>150 ng/ml) (**Figure 9A**), and approximately 55% of the mice induced specific IgM (>150 U/ml) (**Figure 9B**), although the titer of antibodies indicated high fluctuation levels. The total IgG in serum was not significantly increased by αRP105-TM, unlike αRP105 (**Figure S7**). We also analyzed the neutralizing titer of the serum to evaluate its protective effect against the influenza virus. The neutralizing titer obtained from the immunization with HA and αRP105-TM was significantly higher (>6.4-fold) than that from the isotype control (**Figure 9C**). These results demonstrated that αRP105-TM as an adjuvant could induce a partial but significant increase in serum IgG and IgM levels against HA and induce almost neutralizing responses.

A day after obtaining the serum, all mice were infected with a lethal dose of A/PR8 influenza virus. Three days post-infection, bronchoalveolar lavage wash specimens were obtained and assayed for virus titer. The virus titer in the specimens obtained from the immunization with HA and αRP105-TM was significantly lower (7.1-fold) than that from the isotype control (**Figure 9D**). We also immunized and challenged another mouse group as described above and then monitored the changes in body weight and survival rate on day 14. The body weights of all empty vector-administered mice were severely decreased, and all mice were euthanized within 6 days post-infection according to the humane endpoint (**Figure 9E**). Almost 90% of the mice immunized with HA and Isotype-2-TM were also euthanized (**Figure 9F**). On the other hand, approximately 40% of the mice immunized with HA and αRP105-TM survived for more than 14 days with almost no change in body weight. The loss of body weight in some of the mice was delayed by HA and αRP105-TM administration. These results demonstrated that, as an adjuvant, αRP105-TM enhanced the prophylactic effect against a lethal dose of influenza virus infection according to the level of protective antibody response.

## Discussion

Here, we report that αRP105-TM on the cell membrane activates both mature and immature B cells. To our knowledge, the current study indicated, for the first time, that recombinant agonistic mAbs expressed on the cell membrane by gene vector delivery provides adjuvant efficacy for DNA immunization against the influenza virus. In a previous report, Clark and colleagues demonstrated that the adjuvant effect of the anti-RP105 mAb requires a covalently linked antigen (4). To localize anti-RP105 mAb with the membrane antigen HA of the influenza virus on the cell surface, we cloned the antibody gene expressing anti-RP105 mAb and constructed the plasmid encoding anti-RP105 mAb bound to TM. Our current procedure using these vectors is considered to mimic the covalent bonding between anti-RP105 mAb and the antigen because we detected the expression of both αRP105-TM and HA on the cell surface of HEK293T cells. Furthermore, we succeeded in showing that a single gene transfer with the plasmids encoding HA and αRP105-TM significantly induced neutralizing antibodies and provided a prophylactic effect in mice within 14 days after inoculation.

We predict that the liver will be the main target organ for expressing HA and αRP105-TM in the current DNA immunization, since it is well known that the liver synthesizes many serum proteins (54). Previous studies have shown that the hydrodynamics-based procedure in mice can induce gene expression mainly in the liver (29, 30). The level of exogenous expression in the liver was notably higher than that in the other organs. Previously, we potently and rapidly obtained recombinant neutralizing antibodies in the serum by HD with plasmids into mice (31). The liver is a candidate for the target of exogenous expression. Although we could not significantly detect the activation of hepatic B cells in the current study (data not shown), a previous report demonstrated that more than 95% of hepatic B cells express RP105 in the resting state, similar to splenic B cells (55). Other studies have demonstrated that the same number of splenic and hepatic B cells are B220^+^ cells in chronically infected mice (56), and almost the same ratio of immunoglobulins (IgM, IgD, IgG1, and IgG2a), is found on respective B cells (55). Clark and colleagues demonstrated that anti-RP105 mAb bound to the antigen could induce T cell-independent IgG antibody responses in addition to T cell-dependent responses (4). Their results suggest that the anti-RP105 mAb also directly stimulates B cells to produce specific antibodies. From the current results (**Figure 9**) and these studies, we predicted that HD-induced αRP105-TM could interact with hepatic B cells as a target, producing antigen-specific antibodies.

In our previous report (31) and others (29, 30), HD can also induce gene expression in other organs, including the spleen. Clark and colleagues mainly focused on splenic B cells and demonstrated that in B cell–activating factor receptor (BAFFR)-deficient mice, which lack mature B cells but produce T1 B cells (57), anti-RP105 mAb bound to the antigen could also induce the production of antigen-specific antibodies (4, 18). Recently, they also provided an explanation on T1 B cells separated by CD21 and CD24 using flow cytometry analysis (19). Their results also suggest that one of the primary targets of anti-RP105 mAbs is T1 B cells. They also succeeded in protecting BAFFR-deficient mice from a subsequent lethal West Nile virus challenge by vaccination with anti-RP105 mAb bound to the West Nile virus envelope (E) protein (18). In the current study, we also demonstrated that αRP105-TM could stimulate splenic immature B cells, in addition to mature B cells, depending on RP105 (**Figures 6** and **7**). These results suggested that αRP105-TM could also interact with splenic B cells, including immature B cells, to induce antigen-specific antibodies. Further studies are needed to determine which B cells are essential for the current immunization.

To obtain potent antigen-specific antibodies with DNA immunization, it would also be important to determine their expression level and stability in the body. We succeeded in inducing high levels of αRP105 at approximately 10 µg/ml in BALB/c wild-type mice by HD (**Figure 3A**), as previously described (31). The expression level in wild-type mice rapidly decreased 7 days post-HD, whereas it was stable in RP105-KO mice (**Figure 3B**). This result suggested that recombinant antibodies could be retained in the body for a long time unless they interact with the endogenous target, RP105. In a previous study, gene expression by HD with a plasmid expressing luciferase was also transient; that is, the peak expression was reached within 8 h after gene transfer and then rapidly decreased thereafter (29). Another study also demonstrated higher expression levels of recombinant human antibodies in the plasma of SCID mice from HD within a short time after the gene transfer, whereas the level decreased around 45 days later (58); therefore, gene expression after transfer by HD is transient. In addition to other groups, we have previously obtained potent and stable expression of recombinant mAbs for several months using EP into the muscle (28, 58), which is one of the main targets for EP, and can remain long-term extra chromosomally (59, 60). Therefore, EP might induce potent and stable expression of αRP105 and αRP105-TM in the body, followed by an enhancement of its adjuvant efficacy with long-term stimulation of B cells.

Remarkably, the whole IgG level in the serum increased, whereas the expression level of αRP105 rapidly decreased 7 days post-HD, dependent upon RP105 (**Figures 3A, B, C, E**). These results suggest a negative correlation between the level of αRP105 and that of whole IgG in the serum because the consumption of αRP105 induced by HD in the serum seemed to enhance the whole IgG level. Long-term expression of αRP105 induced by another gene transfer method, such as EP, might reveal the causality between the levels of αRP105 and whole IgG.

To obtain stable and potent expression, gene construction is essential. A previous study optimized the stable expression of recombinant mAbs from HC bound to LC *via* F2A elements (33). We also succeeded in enhancing the expression of αRP105-TM on the cell membrane with the F2A element (**Figure 4C**). This bicistronic expression of αRP105-TM has another advantage in that it can simplify the purification of the plasmid during manufacturing, compared with using two plasmids. The 2A peptide, which was derived from the foot-and-mouth disease virus, can undergo self-cleavage and generate two proteins (e.g., full-length antibodies) from a single open reading frame (33, 60). Based on this principle, the 2A-linked genes can generate equal amounts of protein, unlike internal ribosome entry site (IRES)-linked genes, in which the expression of the second gene is significantly lower than the expression of the first gene. To eliminate the 23 residues from self-cleaved 2A remaining at the C terminus in the upstream products, previous designs have added a furin cleavage sequence upstream of 2A (33). In the current study, however, it was suggested that the furin cleavage site did not work because a partial residue of 2A possibly remained in the LC sequence (**Figures S2** and **S4**). A previous study succeeded in eliminating the residues from mAbs using the furin cleavage site in HEK293 cells (33), which we also used. Another group also found that the size of the protein, LC, upstream of F2A, was slightly larger than that of the standard LC, indicating that furin possibly failed to remove the residues of 2A (52). Furin cleavage efficiency depends on the structure of the complete IgG1 monomers due to different V regions, e.g., the efficiency is higher for anti-tumor necrosis factor (TNF)-α and anti-vascular endothelial growth factor (VEGF) than for anti-human epidermal growth factor receptor 2 (HER2), in the LF2AH construction. These results suggest that whether the residues from self-cleaved 2A at the C terminus in the upstream products remain is determined based on each antibody. To avoid the additional residues in Fc, which determine the function of the antibody, the construct of LF2AH would be better than that of HF2AL.

One advantage of gene-based passive immunization is that we can easily modify the construction to obtain a unique antibody (e.g., mAb bound to TM, bicistronic expression) *via* genetic engineering. In the current study, we genetically class-switched the constant region of anti-RP105 mAb from rat IgG2a to mouse IgG1 to avoid unwanted responses in the mice (**Figure 1A**). Unfortunately, the biological activity of αRP105 was much lower than that of the parental anti-RP105 mAb (rat) (**Figure 2**). We and other groups have previously demonstrated that recombinant and parental antibodies have almost the same binding activity to antigens (28, 33). In another one of our studies, we generated constructs of the neutralizing antibodies that have the same variable region but unique respective constant regions (IgG, IgA, IgM, IgD, and IgE) (31). In response to treatment with commercial RDE, which contains sialidase and protease from *Vibrio cholerae* (61), some antibodies (IgG, IgM, and IgA) demonstrated almost the same neutralizing titer against the influenza virus, whereas that of the others (IgD and IgE) were much lower. In our next study, we demonstrated that some glycans on IgE were destroyed by RDE but not by IgG (41). This suggests that the construct of αRP105 could be affected by class switching from rat IgG2a to mouse IgG1, followed by a significant decrease in the binding level. To obtain a higher binding level to RP105, the factor responsible for mouse IgG1 of αRP105 should be identified, in which some of the domains should be exchanged.

Although the current αRP105 reduced the binding level to RP105 compared with the parental anti-RP105 mAb, its biological activity was higher than that of αRP105-TM. αRP105 increased the whole IgG level in serum, whereas αRP105-TM did not (**Figures 3C, E**, and **Figure S7**). αRP105 could also induce higher levels of CD86 on B cells and whole IgG (data not shown). Clark and colleagues have already succeeded in obtaining an extremely potent adjuvant effect of anti-RP105 mAb compared to that of alum (4), a canonical adjuvant (62, 63). However, we are concerned about the safety of the clinical vaccine because anti-RP105 circulates into the whole body *via* blood vessels and could stimulate whole B cells in the body at the same time. If we can obtain enough adjuvant effect with αRP105-TM, it would be better because of localization, rather than secretion (**Figure 4B**). The current gene transfer with HD, which can induce gene expression in several tissues, is also not suitable for clinical trials, although it can easily obtain potent gene expression in mice without a special device. Gene transfer with EP, which is the most popular non-viral system used in clinical trials (64–66), would be more suitable because the effect of αRP105-TM could be localized.

In the current study, we suggested that αRP105-TM is localized on the cell membrane because we could not detect the secreted αRP105-TM in the supernatant from HEK293T cells (**Figure 4B**). Intriguingly, we detected αRP105-TM on B cells co-cultured with αRP105-TM-expressing cells dependent upon RP105 (**Figure 8**). Clark and colleagues previously indicated that RP105 is internalized after ligation by anti-RP105 mAb as unpublished data (4). In the current study, we found that approximately 49.5% of the B220^+^IgD^+^ cells were negative for mIgG1 (**Figure 8A**, left panel, third data from the top). These results suggested that internalization could contribute to reducing the level of RP105 on the membrane of B cells (**Figures 6B** and **7A**, left panel). On the other hand, our results also suggested that αRP105-TM on B cells could contribute to a reduction in the detection levels by masking RP105 and blocking the detection antibodies. We might be able to consider that αRP105-TM transferred from the HEK293T cells to the B cell membrane depends on RP105 through trogocytosis. Several reports have documented one of the cross-dressings between two live cells (e.g., monocytes and anti-CD20-bound B cells), termed trogocytosis (67–69). Lymphocytes can share or exchange membrane and membrane-associated proteins through the immunological synapse within minutes of conjugate formation. In clinical observations, previous reports suggested that trogocytosis corresponds to the loss of CD20 from circulating malignant B cells after the infusion of rituximab, a humanized anti-CD20 mAb (70–72). We might then find a new phenomenon in B cells with stimulation using αRP105-TM because, to our knowledge, stimulation such as trogocytosis *via* RP105 has never been conducted. In addition, we expect to obtain insight into the natural RP105 ligand, which has not yet been identified (5, 6).

In conclusion, an adjuvant effect for DNA immunization against influenza was achieved using a new passive immunization method with αRP105-TM. Gene-based passive immunization is cost-effective because of its high stability and ease of manufacture in microorganisms. Anti-RP105 mAb can respond to immature B cells in addition to mature B cells. Clark and colleagues have already succeeded in inducing potent neutralizing antibodies by a West Nile virus E-anti-RP105 mAb vaccine (18). By further improving the affinity of αRP105-TM, in addition to the advantage of simple preparation of the vaccination, it might induce unique neutralizing antibodies, thereby avoiding induction by conventional vaccines. The induced antibodies could then provide broad cross-protection against influenza virus infection.

## Data Availability Statement

The datasets presented in this study can be found in online repositories. The names of the repository/repositories and accession number(s) can be found in the article/**Supplementary Material**.

## Ethics Statement

The animal study was reviewed and approved by The Animal Research Committee of the Aichi Medical University.

## Author Contributions

TY contributed to the study design, performed most experiments, analyzed and interpreted the data, and wrote and supervised the manuscript. MB performed the partial experiments. KK, MN, HH, and AA provided tools and reagents. MI, ST, HT, II, and FN assisted with the study design and interpreted data. JC assisted with supervision of the manuscript. SA-T contributed to the study design and supervised the manuscript. All authors contributed to the article and approved the submitted version.

## Funding

This review was funded by JSPS KAKENHI Grant Number 19K07491 (to TY), 19K08500 (to SA-T), Takeda Science Foundation (to TY), Daiko Foundation (to SA-T), a grant from the International Joint Usage/Research Center, the Institute of Medical Science, University of Tokyo (ID. no. 2019-3062), and a grant from Unit support, Aichi Medical University (to TY and SA-T).

## Conflict of Interest

The authors declare that the research was conducted in the absence of any commercial or financial relationships that could be construed as a potential conflict of interest.

## Abbreviations

TM, transmembrane domain; HEK293T, human embryonic kidney cells 293 that stably express the SV40 large T antigen; RP105, radioprotective 105; MD-1, myeloid differentiation protein 1; A/PR8, A/Puerto Rico/8/34; HA, hemagglutinin; OVA, chicken oval albumin; V_H_, variable region of heavy chain; HD, hydrodynamic injection; EP, electroporation; RDE, receptor destroying enzyme; HC, heavy chain; LC, light chain; F2A, furin-2A; T1, traditional 1; mAb, monoclonal antibody; TLR, Toll-like receptor; KO, knockout; gMFI, geometric mean fluorescence intensity; αRP105, recombinant anti-RP105 mAb; Isotype-1, recombinant anti-HA mAb; Isotype-2, recombinant anti-OVA mAbs; PFU, plaque-forming units.
