# Supplementary material for: A Novel Gene Delivery Vector of Agonistic Anti-Radioprotective 105 Expressed on Cell Membranes Shows Adjuvant Effect for DNA Immunization Against Influenza
Source: Front Immunol. 2020 Dec 22;11:606518. doi: 10.3389/fimmu.2020.606518 (PMC7783388; doi:10.3389/fimmu.2020.606518)
Supplement: Supplementary file 1 [file DataSheet_1.pdf]

Supplementary Figures

A Novel Gene Delivery Vector of Agonistic Anti-Radioprotective 105 Expressed on Cell Membranes Shows Adjuvant Effect for DNA Immunization Against Influenza

Tatsuya Yamazaki[1,4*], Mrityunjoy Biswas[1], Kouyu Kosugi[2], Maria Nagashima[2], Masanori Inui[1], Susumu Tomono[1], Hidekazu Takagi[1], Isao Ichimonji[1], Fumiaki Nagaoka[1], Akira Ainai[3], Hideki Hasegawa[3], Joe Chiba[2], and Sachiko Akashi-Takamura[1*]

[1]Department of Microbiology and Immunology, School of Medicine, Aichi Medical University, Aichi, Japan

[2]Department of Biological Science and Technology, Tokyo University of Science, Tokyo, Japan

[3]Department of Pathology, National Institute of Infectious Diseases, Tokyo, Japan

***Correspondence:**

Tatsuya Yamazaki

yamazaki13@aichi-med-u.ac.jp

Sachiko Akashi-Takamura

sachiko@aichi-med-u.ac.jp

[4]Lead contact: Tatsuya Yamazaki; E-mail: yamazaki13@aichi-med-u.ac.jp

Supplementary Figure 1

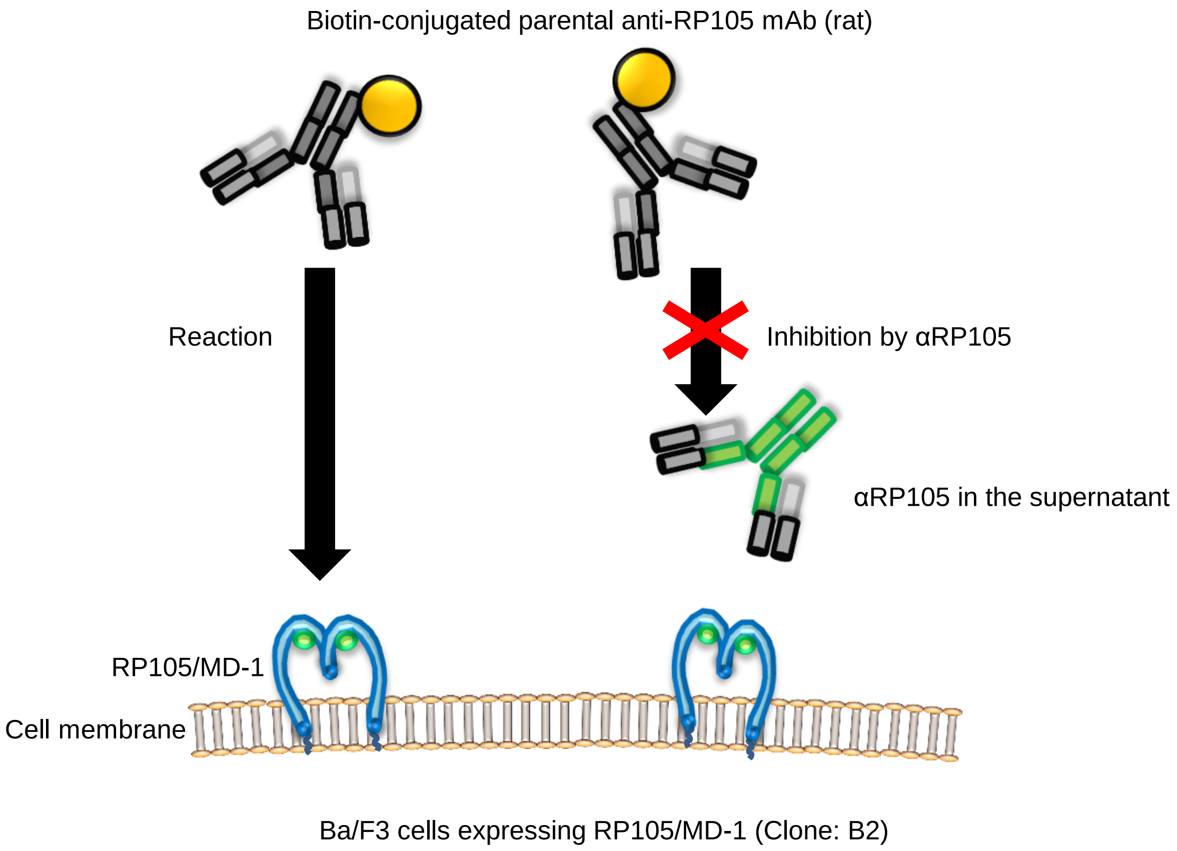

Supplementary Figure 1| Scheme of the competitive binding assay method. The Ba/F3 cells expressing RP105/MD-1 (Clone: B2) were incubated with the parental anti-RP105 mAb (rat) or the supernatants obtained from the HEK293T cells, followed by incubation with biotinylated parental anti-RP105 mAb (rat). The binding level was analyzed with geometric mean fluorescence (gMFI) using a BD LSRFortessa.

Supplementary Figure 2

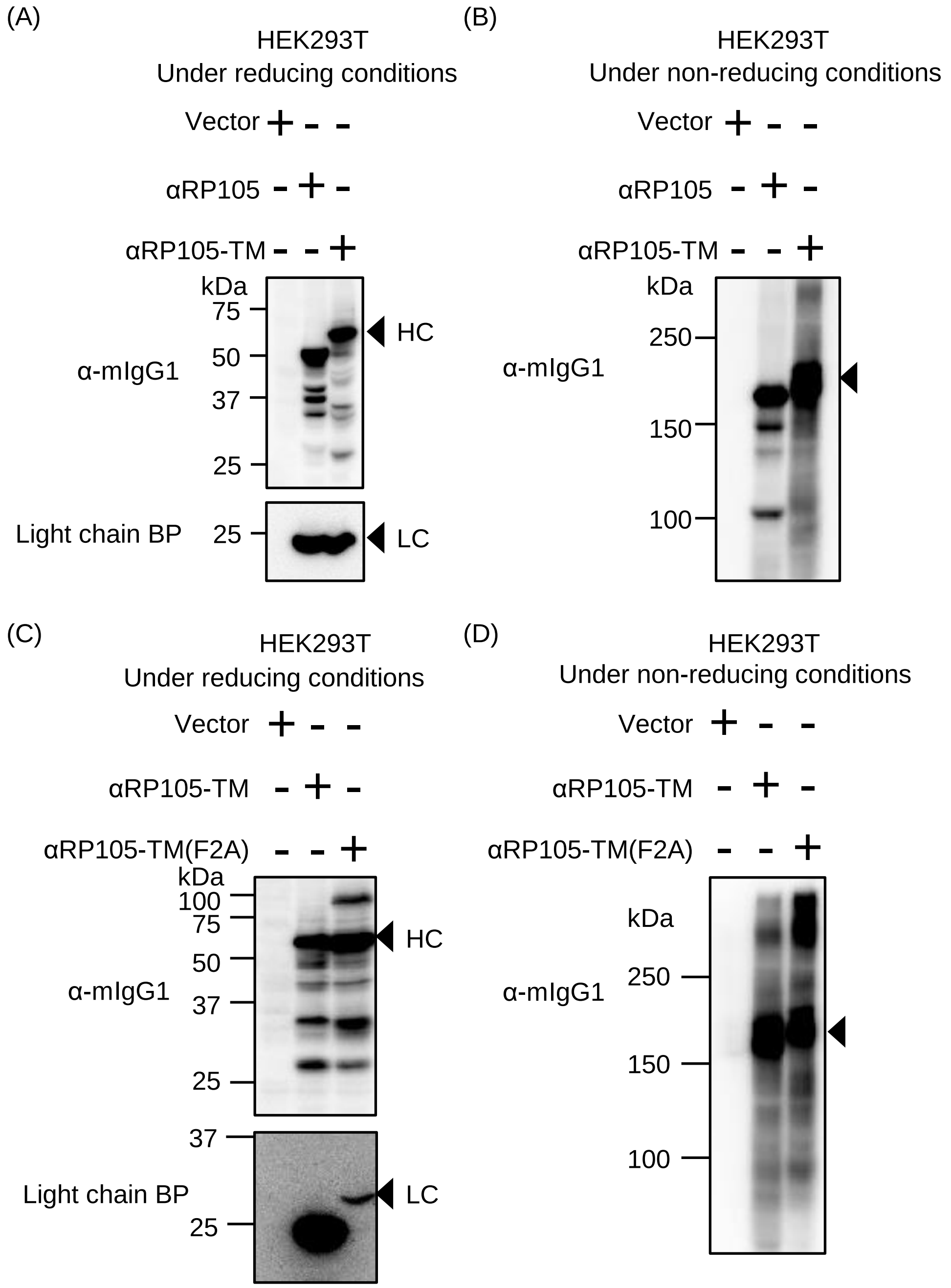

Supplementary Figure 2| *In vitro* expression of αRP105-TM and αRP105-TM (F2A). (A, B) HEK293T cells were transfected with pCADEST1-empty (Vector), pCADEST1-anti-RP105 mIgG1 and pCADEST1-anti-RP105 mkappa (αRP105), or pCADEST1-anti-RP105 mIgG1-TM and pCADEST1-anti-RP105 mkappa (αRP105-TM). Two days later, the cell lysates were collected and processed by western blotting under reducing **(A)** or non-reducing **(B)** conditions, followed by probing with HRP-conjugated goat anti-mouse IgG1 and HRP-conjugated light chain-binding protein (BP). **(C, D)** HEK293T cells were transfected with pCADEST1-empty (Vector), pCADEST1-anti-RP105 mIgG1-TM and pCADEST1-anti-RP105 mkappa (αRP105-TM), or pCADEST1-anti-RP105 kappa-F2A-anti-RP105 mIgG1-TM [αRP105-TM (F2A)]. Two days later, the cell lysates were collected and processed by western blotting under reducing **(C)** or non-reducing **(D)** conditions, followed by probing with HRP-conjugated goat anti-mouse IgG1 and HRP-conjugated light chain-BP. All indicated data are representative of two independent experiments.

Supplementary Figure 3

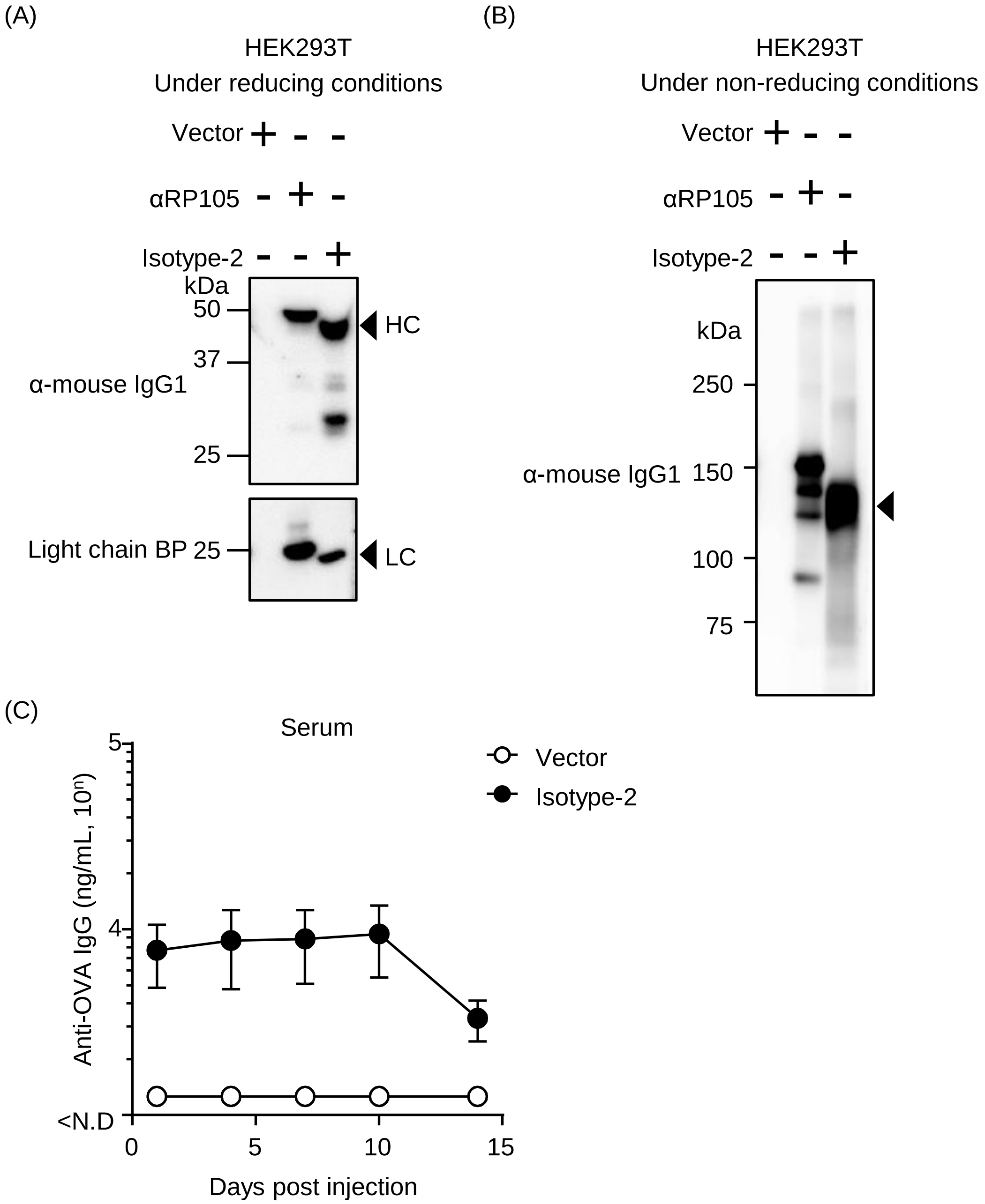

Supplementary Figure 3| *In vitro* and *in vivo* expression of recombinant anti-OVA mIgG1 (Isotype-2). (A, B) HEK293T cells were transfected with pCADEST1(Vector), pCADEST1-anti-RP105 mIgG1 and pCADEST1-anti-RP105 mkappa (αRP105), or pCADEST1-anti-OVA mIgG1 and pCADEST1 anti-OVA mkappa (Isotype-2). Seven days later, the supernatant was obtained and processed by western blotting under reducing **(A)** or non-reducing **(B)** conditions, followed by probing with HRP-conjugated goat anti-mouse IgG1 or HRP-conjugated light chain-binding protein (BP). **(C)** BALB/c mice (n = 5) were subjected to HD with the pCADEST1 (Vector) or pCADEST1-anti-OVA mIgG1 and pCADEST1 anti-OVA mkappa (Isotype-2). The serum was obtained at the indicated time, and the level of anti-OVA IgG was analyzed by quantitative ELISA. The detection limit was over 1230 (=$10^{3.09}$) ng/mL. Data are represented as mean ± S.D. All indicated data are representative of two independent experiments.

Supplementary Figure 4

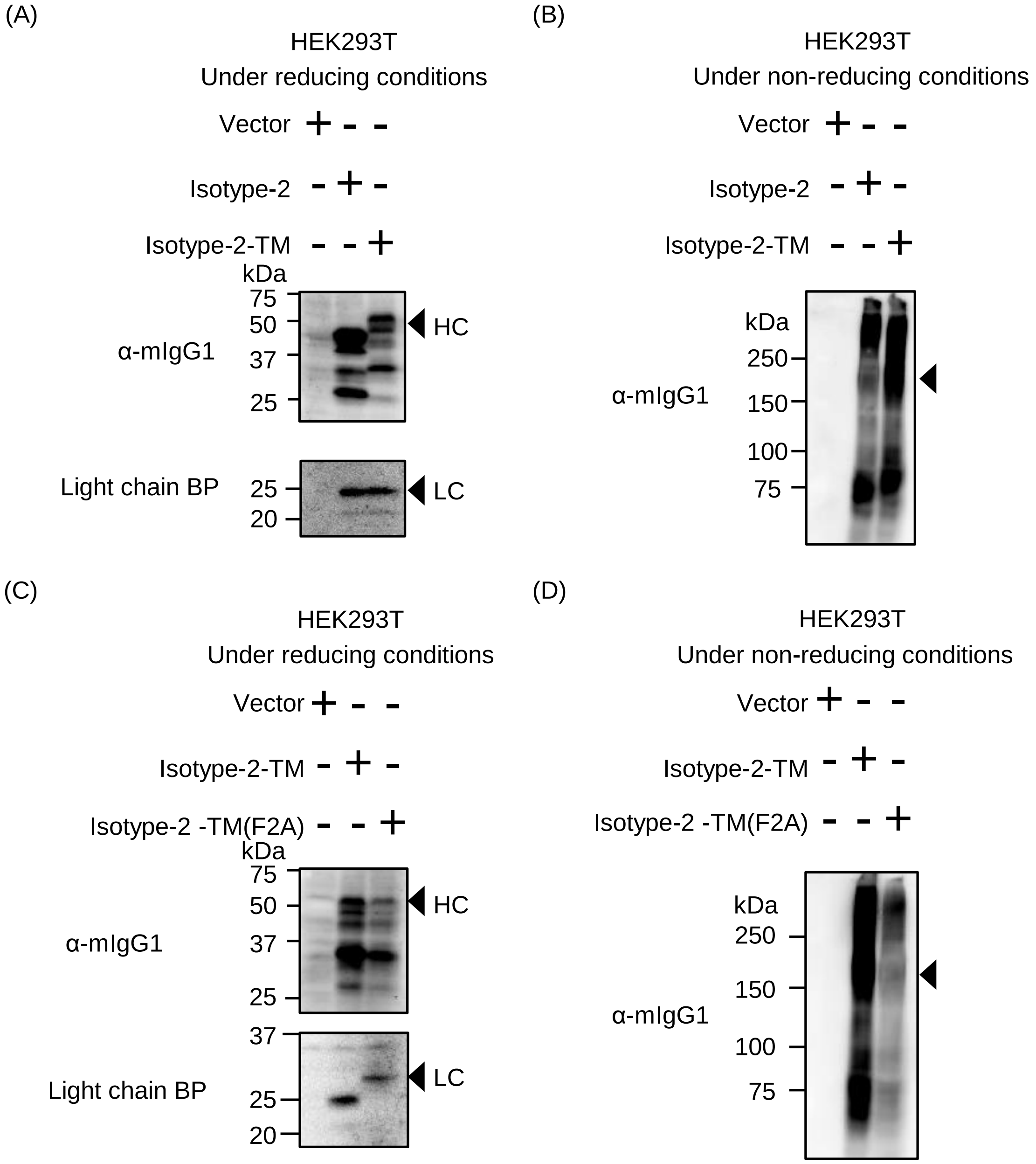

Supplementary Figure 4| ***In vitro*** **expression of Isotype-2-TM. (A-D)** HEK293T cells were transfected with pCADEST1-empty (Vector), pCADEST1-anti-OVA mIgG1 and pCADEST1-anti-OVA mkappa (Isotype-2), pCADEST1-anti-OVA mIgG1-TM and pCADEST1-anti-OVA mkappa (Isotype-2-TM) **(A, B)**, or pCADEST1-anti-OVA kappa-F2A-anti-OVA mIgG1-TM [Isotype-2-TM (F2A)] **(C, D)** as indicated. Two days later, cells were lysed with lysis buffer containing 2% SDS and processed by western blotting under reducing **(A, C)** or non-reducing **(B, D)** conditions, followed by probing with HRP-conjugated goat anti-mouse IgG1 and HRP-conjugated light chain-binding protein (BP). All indicated data are representative of two independent experiments.

Supplementary Figure 5

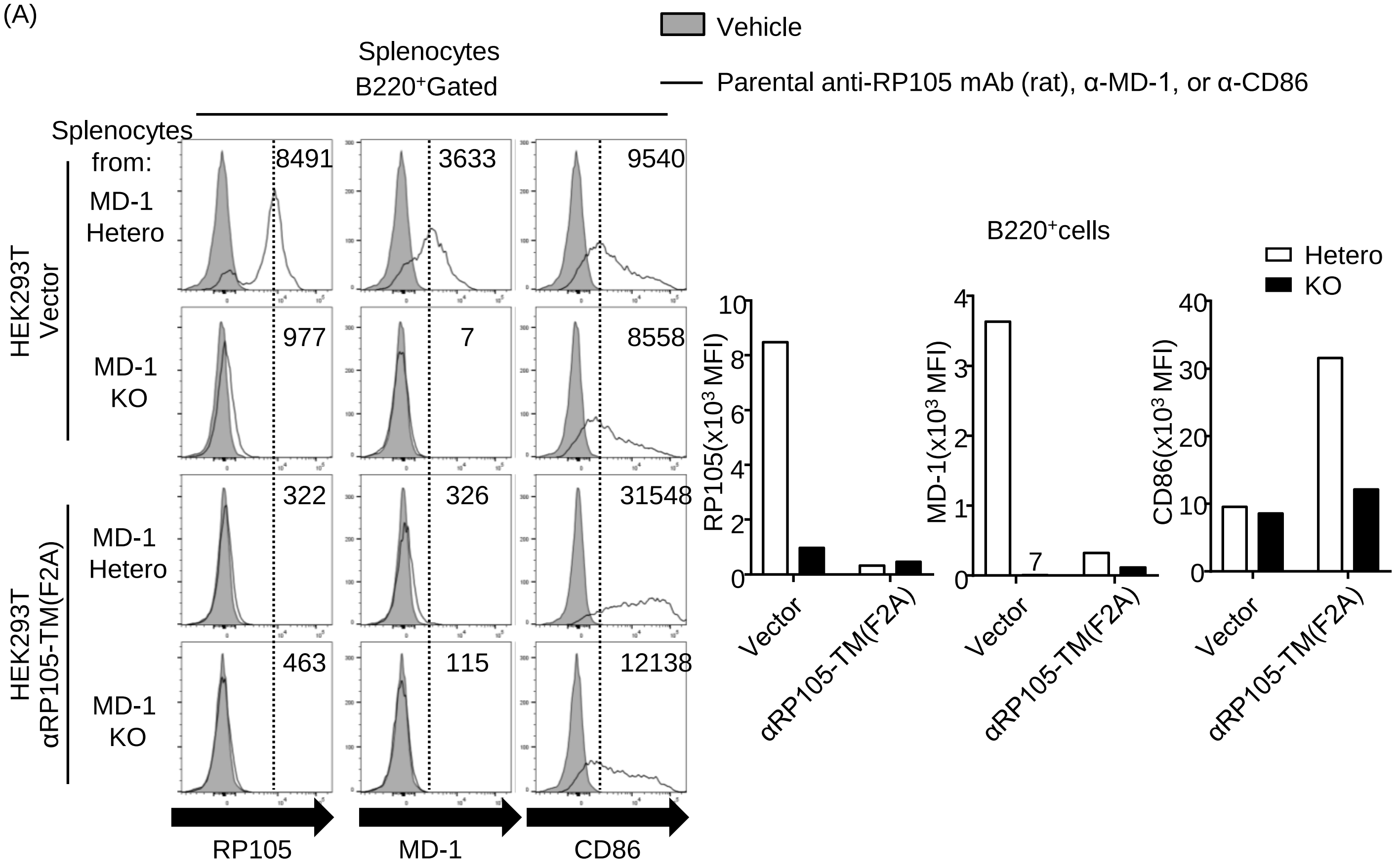

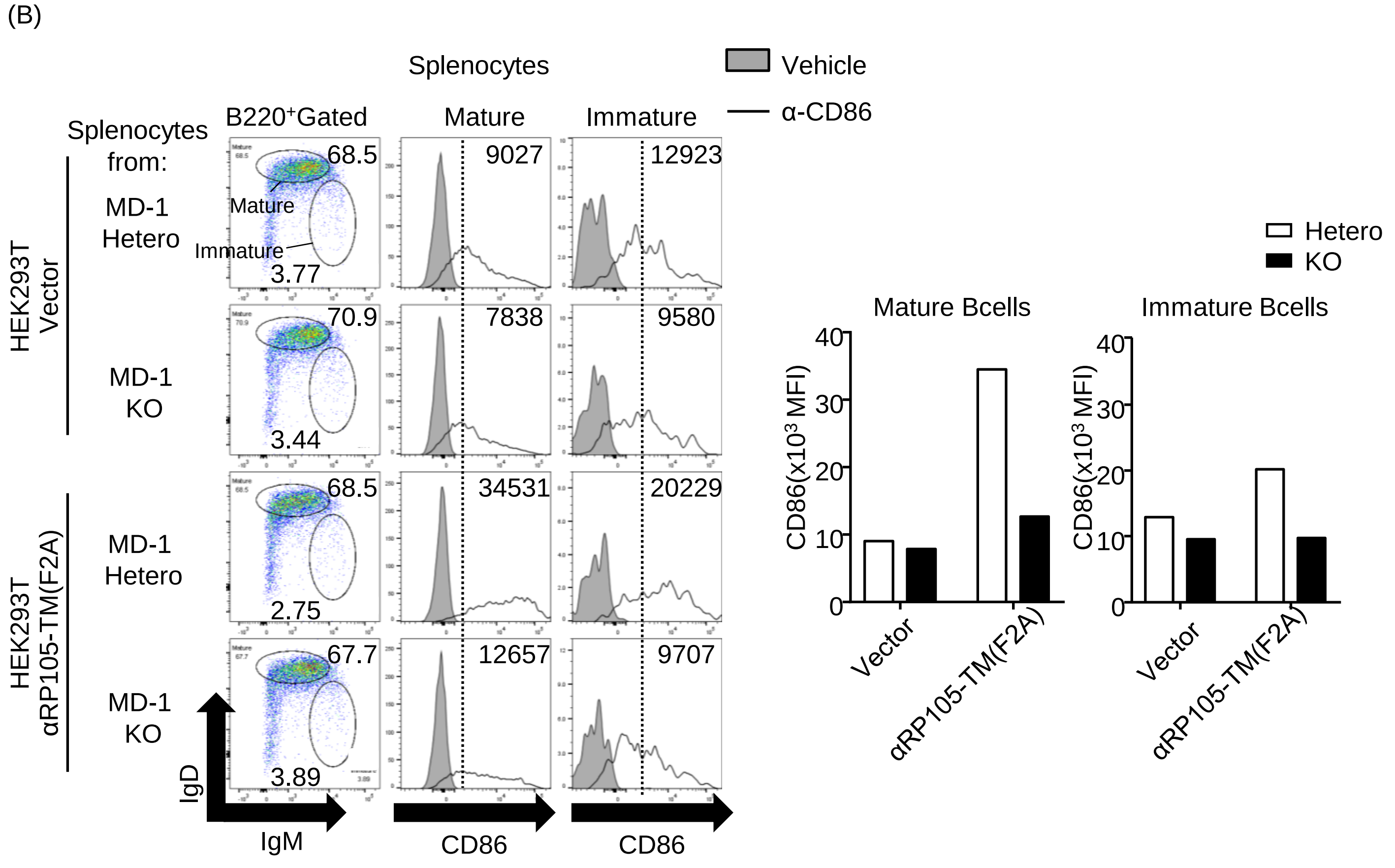

Supplementary Figure 5

Supplementary Figure 5| αRP105-TM can activate B cells that depends on RP105 expression on the cell membrane. (A, B) HEK293T cells were transfected with pCADEST1-empty (Vector) or pCADEST1-anti-RP105 kappa-F2A-anti-RP105 mIgG1-TM [αRP105-TM (F2A)]. Two days later, the splenocytes obtained from MD-1-Hetero and MD-1-KO BALB/c mouse were co-cultured with HEK293T cells for two days. Then, the splenocytes, which were gated on B220 **(A)**, IgM^{low} IgD^{high} as mature B cells (Middle panel in **B**) or IgM^{high} IgD^{low} as immature B cells (Right panel in **B**), were respectively incubated with biotinylated parental anti-RP105 mAb (rat), anti-MD-1, or anti-CD86 antibodies as indicated, followed by incubation with PE-conjugated streptavidin. The indicated numbers on each gate (Left panel in **B**) respectively represent the percentage of cells of mature or immature B cells gated on $B220^{+}$ cells. The numbers in the histogram represent MFI. The expression level was analyzed using a BD LSRFortessa. All indicated data are representative of two independent experiments.

Supplementary Figure 6

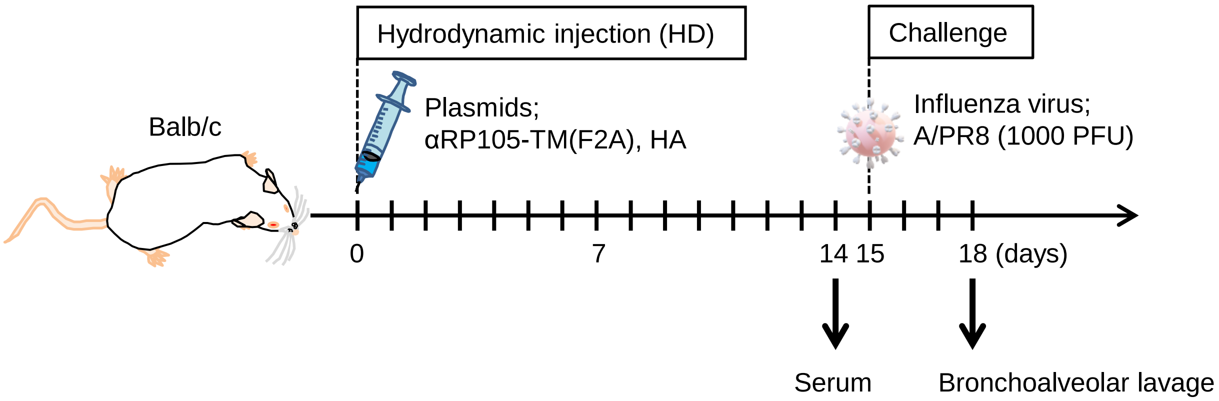

Supplementary Figure 6|The schedule of the immunization and challenge of Figure 9.

Supplementary Figure 7

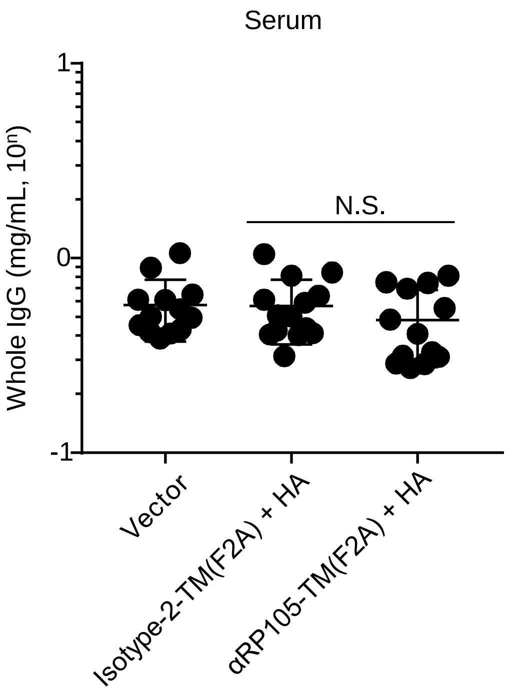

Supplementary Figure 7| The whole IgG level is not significantly increased by αRP105-TM. Whole IgG levels in the serum from **Figure 9** were analyzed by quantitative ELISA. The detection limit was over 0.0147 (=$10^{-1.832}$) mg/mL. The indicated data are combined from two independent experiments (n = 13–14) and indicated as the mean ± S.D. N.S., not significant (Mann-Whitney test).

Supplementary Figure 8

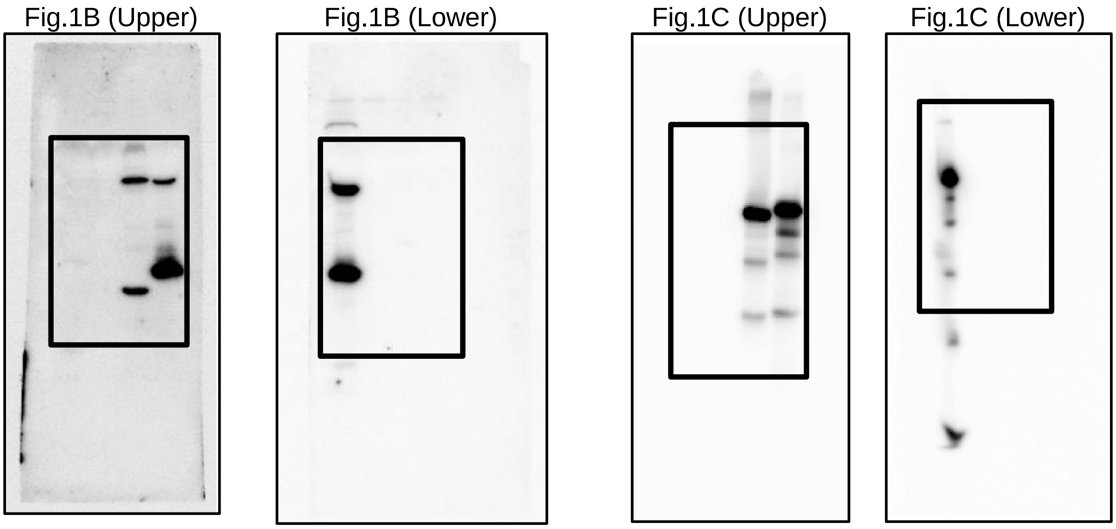

Supplementary Figure 8| Full-length blots from the primary figures. The cropped areas used in the current article are indicated in black boxes.

Supplementary Figure 9

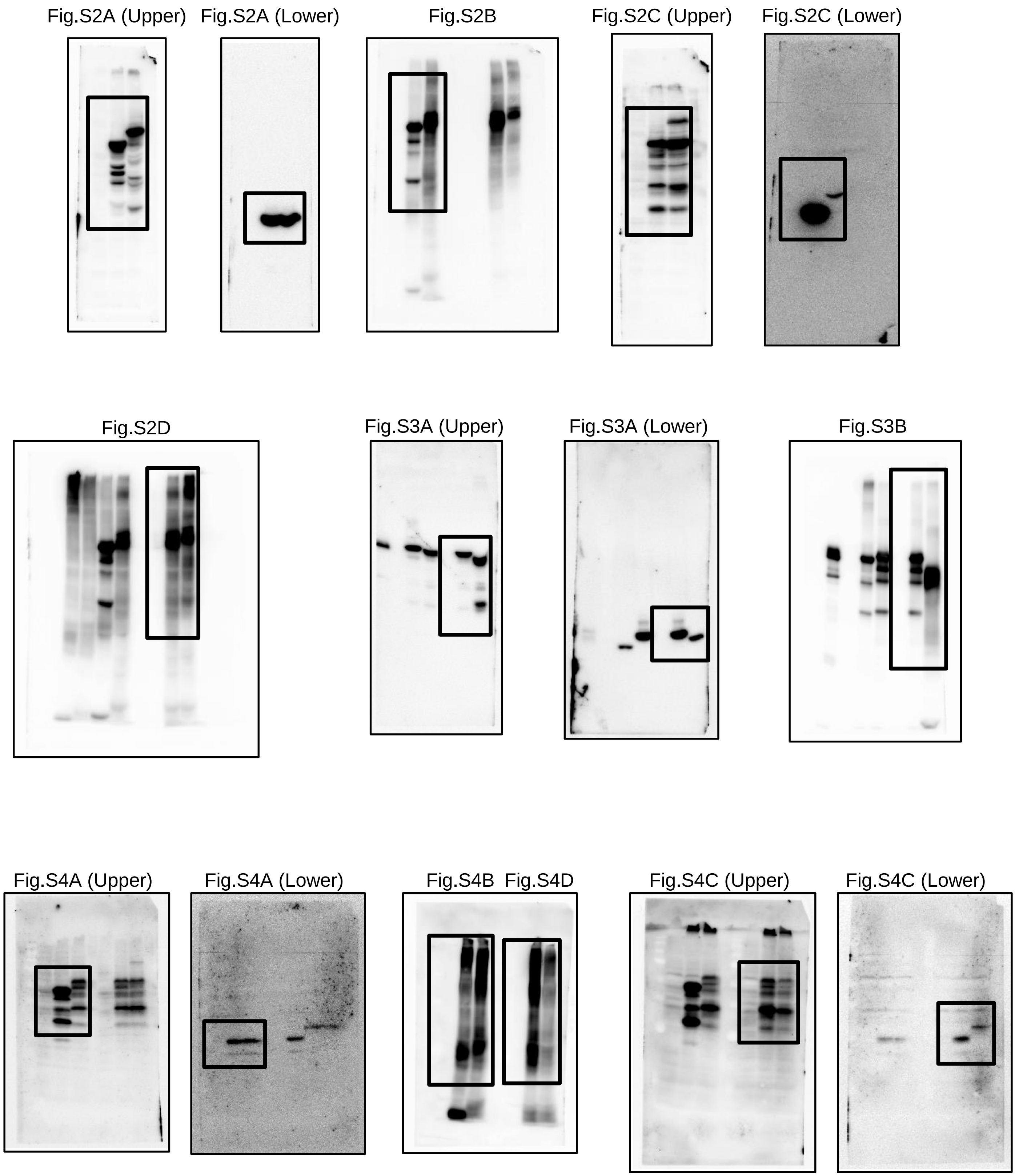

Supplementary Figure 9| Full-length blots from the supplementary figures. The cropped areas used in the current article are indicated in black boxes.

Supplementary Table 1 | The following primers were used for cloning indicated constructions.

**Primer detail**	**Primer sequence**	**Construction**
Anti-RP105 V_H Forward	5'-atGCGGCCGCATGGAACTGGGTCTTTTTTTCC-3'	pCADEST1-anti-RP105 mIgG1
Mouse IgG1-overlapped anti-RP105 V_H Reverse	5'-GATGGGGGTGTCGTTTTGGCTGTTGTTTCAGCTGAGGAGA-3'	pCADEST1-anti-RP105 mIgG1
Mouse IgG1 Forward	5'-GCCAAAACGACACCCCCATC-3'	pCADEST1-anti-RP105 mIgG1
Mouse IgG1 Reverse	5'-aGGCGCGCCTCATTTACCAGGAGAGTGGG-3'	pCADEST1-anti-RP105 mIgG1
Anti-RP105 V_L Forward	5'-aGCGGCCGCATGGAGACAGACAGACTCCT-3'	pCADEST1-anti-RP105 mkappa
Anti-RP105 V_L Reverse	5'-TTTCAACTCCAGCTTGGTGC-3'	pCADEST1-anti-RP105 mkappa
Anti-RP105 V_L-overlapped mouse kappa Forward	5'-GCACCAAGCTGGAGTTGAAACGGGCTGATGCTGCACCAAC-3'	pCADEST1-anti-RP105 mkappa
Mouse kappa Reverse	5'-aGGCGCGCCCTAACACTCATTCCTGTTGA-3'	pCADEST1-anti-RP105 mkappa
Anti-OVA V_H Forward	5'-atGCGGCCGCATGGCCGTACTCGGCCTCTT-3'	pCADEST1-anti-OVA mIgG1
Mouse IgG1-overlapped anti-OVA V_H Reverse	5'-GATGGGGGTGTCGTTTTGGCAGACACTGTGACCAGTGTTC-3'	pCADEST1-anti-OVA mIgG1
A/PR8-HA Forward	5'-atGCGGCCGCATGAAGGCAAACCTACTGGT-3'	pCADEST1-HA
A/PR8-HA Reverse	5'-aGGCGCGCCTCAGATGCATATTCTGCACT-3'	pCADEST1-HA
