# Supplementary material for: A Novel Gene Delivery Vector of Agonistic Anti-Radioprotective 105 Expressed on Cell Membranes Shows Adjuvant Effect for DNA Immunization Against Influenza
Source: Front Immunol. 2020 Dec 22;11:606518. doi: 10.3389/fimmu.2020.606518 (PMC7783388; doi:10.3389/fimmu.2020.606518)
Supplement: Supplementary file 2 [file DataSheet_2.pdf]

Supplementary Data

A Novel Gene Delivery Vector of Agonistic Anti-Radioprotective 105 Expressed on Cell Membranes Shows Adjuvant Effect for DNA Immunization Against Influenza

Tatsuya Yamazaki[1,4*], Mrityunjoy Biswas[1], Kouyu Kosugi[2], Maria Nagashima[2], Masanori Inui[1], Susumu Tomono[1], Hidekazu Takagi[1], Isao Ichimonji[1], Fumiaki Nagaoka[1], Akira Ainai[3], Hideki Hasegawa[3], Joe Chiba[2], and Sachiko Akashi-Takamura[1*]

[1]Department of Microbiology and Immunology, School of Medicine, Aichi Medical University, Aichi, Japan

[2]Department of Biological Science and Technology, Tokyo University of Science, Tokyo, Japan

[3]Department of Pathology, National Institute of Infectious Diseases, Tokyo, Japan

***Correspondence:**

Tatsuya Yamazaki

yamazaki13@aichi-med-u.ac.jp

Sachiko Akashi-Takamura

sachiko@aichi-med-u.ac.jp

[4]Lead contact: Tatsuya Yamazaki; E-mail: yamazaki13@aichi-med-u.ac.jp

Supplementary Data|

We indicate amino acid sequences of αRP105-TM (F2A), Isotype-2-TM (F2A), and HA (A/PR8). The signal sequence was determined by SignalP-5.0 Server (http://www.cbs.dtu.dk/services/SignalP/). The variable region, constant region, and trans membrane part were determined by ImMunoGeneTics (IMGT, http://www.imgt.org/).

Amino acid sequence of αRP105-TM (F2A)

METDRLLLWVLLLWVPGSTGDTVLTQSPTLAVSPGERVSISCRASEGVNSYMHWYLQRPG 60

QQPKLLIYKASNLASGVPARFSGSGSGTDFTLTIDPVEADDTATYFCQQSWNDPRTFGGG 120

TKLELKRADAAPTVSIFPPSSEQLTSGGASVVCFLNNFYPKDINVKWKIDGSERQNGVLN 180

SWTDQDSKDSTYSMSSTLTLTKDEYERHNSYTCEATHKTSTSPIVKSFNRNECRAKRAPV 240

KQTLNFDLLKLAGDVESNPGPRGRMELGLFFLLSVTAEVHSQVQLQQSGAELAKPGSSVK 300

ISCKASGYTFTTDYISWIKQTTGQGLEYIGYIITAGGGTNYNEKFKGKATLTVDKSSSTA 360

FMQLSSLTPDDSAVYYCARGRIRTLDFDYWGQGVMVTVSSAETTAKTTPPSVYPLAPGSA 420

AQTNSMVTLGCLVKGYFPEPVTVTWNSGSLSSGVHTFPAVLQSDLYTLSSSVTVPSSTWP 480

SETVTCNVAHPASSTKVDKKIVPRDCGCKPCICTVPEVSSVFIFPPKPKDVLTITLTPKV 540

TCVVVDISKDDPEVQFSWFVDDVEVHTAQTQPREEQFNSTFRSVSELPIMHQDWLNGKEF 600

KCRVNSAAFPAPIEKTISKTKGRPKAPQVYTIPPPKEQMAKDKVSLTCMITDFFPEDITV 660

EWQWNGQPAENYKNTQPIMDTDGSYFVYSKLNVQKSNWEAGNTFTCSVLHEGLHNHHTEK 720

SLSHSPLQLDETCAEAQDGELDGLWTTITIFISLFLLSVCYSAAVTLFKVKWIFSSVVEL 780

KQTLVPEYKNMIGQAP*

Dotted line: Signal sequence

Double line (Blue): Variable region (V_L), Bold line (Blue): Constant region (mkappa)

Double line (Green): Variable region (V_H), Bold line (Green): Constant region (mIgG1)

Shaded characters: F2A sequence

Enclosed character: Trans membrane part

*: Stop codon

Amino acid sequence of Isotype-2-TM (F2A)

MVLMLLLLWVSGTCGDIVMSQSPSSLAVSVGEKVTMSCKSSQSLLFGSNQKNSLAWYQQK 60

PGQSPKLLIYWASTRESGVPDRFTGSGSGTDFTLTISSVKAEDLAVYYCQQYYSYPYTFG 120

GVTKLEIKRADAAPTVSIFPPSSEQLTSGGASVVCFLNNFYPKDINVKWKIDGSERQNGV 180

LNSWTDQDSKDSTYSMSSTLTLTKDEYERHNSYTCEATHKTSTSPIVKSFNRNECRAKRA 240

PVKQTLNFDLLKLAGDVESNPGPRGRMAVLGLFLCLVTFPSCVLSQVQLKQSGPGLVQPS 300

QSLSITCTVSGFSLTSYGIHWVRQSPGKGLEWLGVIWTGGNTVYNAAFISRLSITKDNSK 360

SQVFFKMNSLQATDTAFYYCARNWDYGYDWFAYWGQGTLVTVSAKTTPPSVYPLAPGSAA 420

QTNSMVTLGCLVKGYFPEPVTVTWNSGSLSSGVHTFPAVLQSDLYTLSSSVTVPSSTWPS 480

ETVTCNVAHPASSTKVDKKIVPRDCGCKPCICTVPEVSSVFIFPPKPKDVLTITLTPKVT 540

CVVVDISKDDPEVQFSWFVDDVEVHTAQTQPREEQFNSTFRSVSELPIMHQDWLNGKEFK 600

CRVNSAAFPAPIEKTISKTKGRPKAPQVYTIPPPKEQMAKDKVSLTCMITDFFPEDITVE 660

WQWNGQPAENYKNTQPIMDTDGSYFVYSKLNVQKSNWEAGNTFTCSVLHEGLHNHHTEKS 720

LSHSPLQLDETCAEAQDGELDGLWTTITIFISLFLLSVCYSAAVTLFKVKWIFSSVVELK 780

QTLVPEYKNMIGQAP*

Dotted line: Signal sequence

Double line (Blue): Variable region (V_L), Bold line (Blue): Constant region (mkappa)

Double line (Green): Variable region (V_H), Bold line (Green): Constant region (mIgG1)

Shaded characters: F2A sequence

Enclosed character: Trans membrane part

*: Stop codon

Amino acid sequence of HA (A/PR8)

MKANLLVLLCALAAADADTICIGYHANNSTDTVDTVLEKNVTVTHSVNLLEDSHNGKLCR 60

LKGITPLQLGNCNIAGWLLGNPECDPLLPVRSWSYIVETPNSENGICYPGDFIDYEELRE 120

QLSSVSSFERFEIFPKESSWPNHNTNKGVTAACSHAGKSSFYRNLLWLTEKEGSYPKLKN 180

SYVNKKGKEVLVLWGIHHPSNSKEQQNLYQNENAYVSVVTSNYNRRFTPEIAERPKVKDQ 240

AGRMNYYWTLLKPGDTIIFEANGNLIAPRYAFALSRGFGSGIITSNASMHECNTKCQTPL 300

GAINSSLPFQNIHPVTIGECPKYVRSAKLRMVTGLRNIPSIQSRGLFGAIAGFIEGGWTG 360

MIDGWYGYHHQNEQGSGYAADQKSTQNAINGITNKVNSVIEKMNTQFTAVGKEFNKLEKR 420

MENLNKKVDDGFLDIWTYNAELLVLLENERTLDFHDSNVKNLYEKVKSQLKNNAKEIGNG 480

CFEFYHKCDNECMESVRNGTYDYPKYSEESKLNREKVDGVKLESMGIYQILAIYSTVASS 540

LVLLVSLGAISFWMCSNGSLQCRICI*

Dotted line: Signal sequence

*: Stop codon
